# Reactive oxygen species mediate conical cell shaping in *Arabidopsis thaliana* petals

**DOI:** 10.1371/journal.pgen.1007705

**Published:** 2018-10-08

**Authors:** Xie Dang, Peihang Yu, Yajun Li, Yanqiu Yang, Yu Zhang, Huibo Ren, Binqinq Chen, Deshu Lin

**Affiliations:** 1 College of Life Science, Fujian Provincial Key Laboratory of Haixia Applied Plant Systems Biology, Basic Forestry and Proteomics Research Center, Fujian Agriculture and Forestry University, Fuzhou, China; 2 Haixia Institute of Science and Technology, Fujian Agriculture and Forestry University, Fuzhou, China; The University of North Carolina at Chapel Hill, UNITED STATES

## Abstract

Plants have evolved diverse cell types with distinct sizes, shapes, and functions. For example, most flowering plants contain specialized petal conical epidermal cells that are thought to attract pollinators and influence light capture and reflectance, but the molecular mechanisms controlling conical cell shaping remain unclear. Here, through a genetic screen in *Arabidopsis thaliana*, we demonstrated that loss-of-function mutations in *ANGUSTIFOLIA* (*AN*), which encodes for a homolog of mammalian CtBP/BARs, displayed conical cells phenotype with wider tip angles, correlating with increased accumulation of reactive oxygen species (ROS). We further showed that exogenously supplied ROS generated similar conical cell phenotypes as the *an* mutants. Moreover, reduced endogenous ROS levels resulted in deceased tip sharpening of conical cells. Furthermore, through enhancer screening, we demonstrated that mutations in *katanin* (*KTN1*) enhanced conical cell phenotypes of the *an-t1* mutants. Genetic analyses showed that AN acted in parallel with KTN1 to control conical cell shaping. Both increased or decreased ROS levels and mutations in *AN* suppressed microtubule organization into well-ordered circumferential arrays. We demonstrated that the AN-ROS pathway jointly functioned with KTN1 to modulate microtubule ordering, correlating with the tip sharpening of conical cells. Collectively, our findings revealed a mechanistic insight into ROS homeostasis regulation of microtubule organization and conical cell shaping.

## Author summary

Plants have diverse cell types with distinct sizes, shapes, and functions. For example, most flowering plants contain specialized conical petal epidermal cells that are thought to attract bee pollinators, but the molecular mechanisms controlling conical cell shaping remain unclear. Here, through a genetic screen in *Arabidopsis thaliana*, we demonstrated that loss-of-function mutations in *ANGUSTIFOLIA* (*AN*), which encodes a homolog of mammalian CtBP/BARs, resulted in swollen conical cells, correlating with increased accumulation of reactive oxygen species (ROS). Furthermore, through an enhancer screening, we demonstrated that mutations in *katanin* (*KTN1*) enhanced conical cell phenotypes of the *an-t1* mutants. Genetic analyses showed that AN acted in parallel with KTN1 to control conical cell shaping. We demonstrated that the AN-ROS pathway jointly functioned with KTN1 to modulate microtubule organization, correlating with the tip sharpening of conical cells. Collectively, our findings revealed a mechanistic insight into ROS-mediated regulation of conical cell shaping.

## Introduction

Plants have evolved diverse epidermal cell types, that differ in size, morphology, and function, to adapt to life on land [1,2]. The *Arabidopsis thaliana* (*A*. *thaliana*) epidermis contain several well-known cell types, including interlocking jigsaw puzzle-shaped leaf pavement cells, tubular root hairs, and elongated hair-like trichomes. Using these three cell types as models to study cell shape, considerable progress has been made over the past two decades toward understanding how plant cells achieve their final morphologies [3,4]. Although similar molecular mechanisms controlling cell shape are shared between these cell types, certain mechanisms are used in a cell-type specific manner [2]. Despite this progress, the molecular mechanisms underlying plant cell shape formation remain to be further explored in specialized cell types.

Conical-shaped cells are specialized epidermal cells decorated with radiate cuticular nanoridges and are usually found in the petal adaxial epidermis of most flowering plants [5–8]. Conical cells are thought to influence light capture and reflectance, temperature, and wettability, and are proposed to have a role in attracting bee pollinators by providing tactile cues [5,9–11]. However, the mechanisms underlying conical cell shaping have remained largely unknown. Most cell types in plants undergo anisotropic cell expansion that is driven by turgor pressure throughout the entire cell surface [12], whereas the direction of expansion and the final cell shape largely depend on the patterning of cell wall architecture through the deposition and the orientation of cellulose microfibrils [13]. Cortical microtubules play an important role in plant cell shape determination by guiding cellulose microfibrils deposition in the cell wall [14–18]. It has been proposed that the co-alignment of circumferential microtubule arrays and cellulose microfibrils restrict cell expansion in girth and promote anisotropic cell expansion perpendicular to the orientation of cellulose microfibrils [14,19]. Genetic and live imaging studies have provided significant progress about the mechanisms underlying the organization of microtubule arrays [20].

We have recently established a confocal microscopy-based imaging approach to study the morphogenesis of conical cells and microtubule organization patterns in these cells [21]. Adaxial epidermal cells in the petal blades from flower development stage 8 are flat [21,22], and undergo tip sharpening after stage 9, which is correlated with the re-orientation of microtubules from disordered to well-ordered circumferential arrays at late developmental stages. Pharmacological evidence demonstrated that normal conical cell expansion requires an intact microtubule network [21]. Despite this progress, it remained elusive how cortical microtubule arrays are organized, and which upstream components are involved during conical cell development.

The *A*. *thaliana ANGUSTIFOLIA* (*AN*) gene, encoding the plant homolog of the C-terminal binding protein/brefeldin A-ADP ribosylated substrate (CtBP/BARS), has been shown to function in microtubule organization, vesicle budding from the Golgi, leaf pavement cell morphogenesis, and trichome branching [23–25], but the underlying molecular mechanisms remain to be explored. In this study, through a reverse genetic screen, we showed that the *AN* knockout mutants *an-t1* and *an-t2* suppressed the tip sharpening of conical cells, correlating with the accumulation of significantly higher levels of reactive oxygen species (ROS). ROS are known for their function as signaling molecules in plant organ growth and development and normal cellular processes [26–29]. Furthermore, mutations in *KTN1*, encoding the p60 microtubule-severing protein [30–33], caused enhanced conical cell phenotypes of *an-t1*. AN acted in parallel with KTN1 to control conical cell tip sharpening. Together, our results suggest that the AN-ROS pathway jointly functions with KTN1 to modulate microtubule ordering and conical cell shaping.

## Results

### Loss of AN function causes wide-angled tips of conical cells, correlating with increased accumulation of ROS

**Fig 1:**
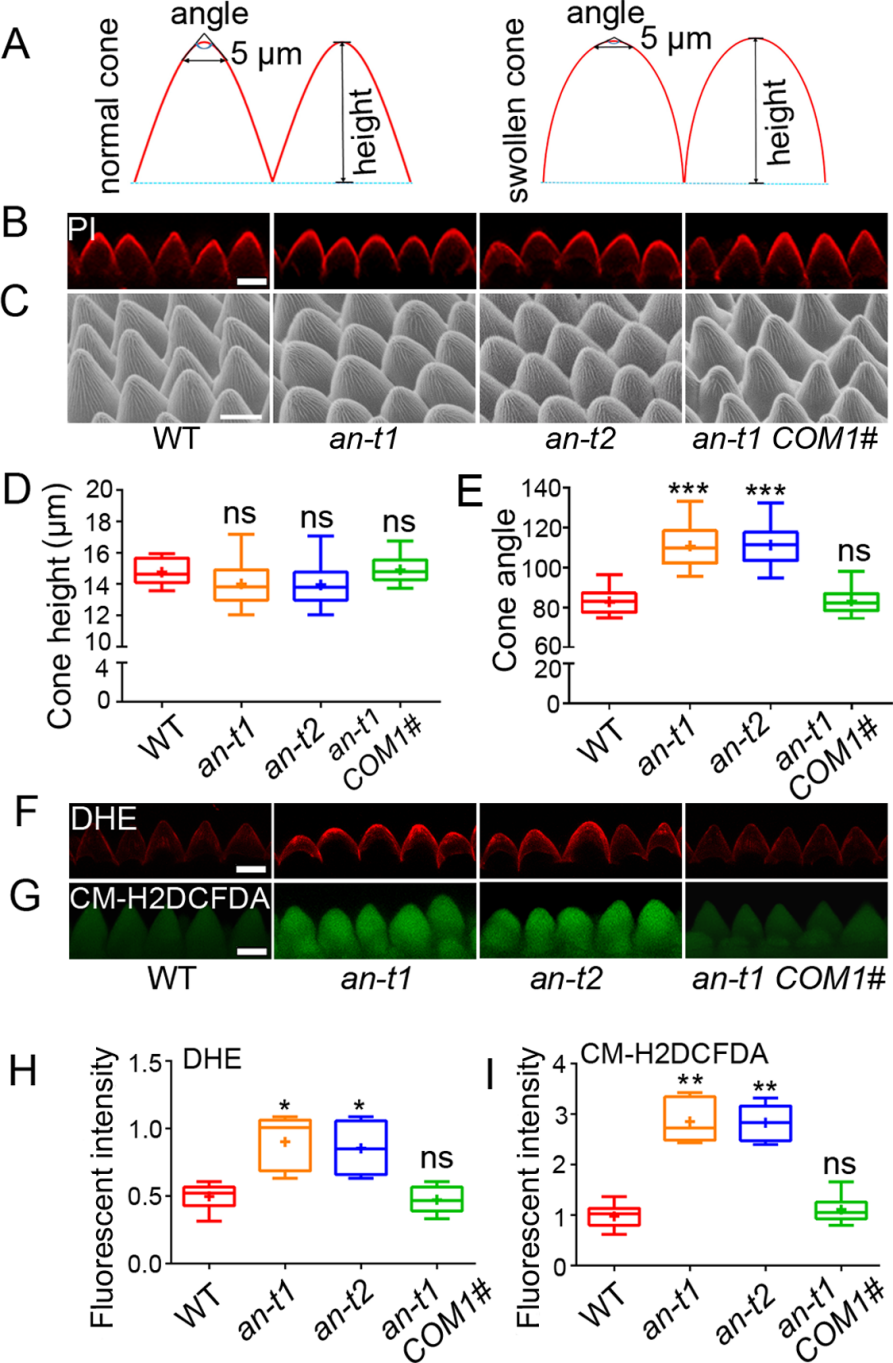
Loss of AN function causes swollen conical cells, correlating with high levels of ROS. (A) A cartoon depicting how cone angle is measured. For both WT and the more dome-like mutants, a straight line of 5 μm is drawn within two side walls of a cell. This straight line is defined as the base, and then other two straight lines are drawn along the two side walls of the cell to form a triangle. The top angle of the triangle is defined as the cone angle of a conical cell. (B) Confocal images of conical cells of WT, *an-t1*, *an-t2*, and *an-t1 COM1#* from stage 14 flowers. Petal blades were folded back, allowing for the side view of conical cells, and stained with a solution containing 10 μg/ml propidium iodide (PI). Scale bar represents 10 μm. (C) Scanning electron microscope images of conical cells of WT, *an-t1*, *an-t2*, and *an-t1 COM1#* from stage 14 flowers. Scale bar represents 10 μm. (D and E) Quantification of cone height (D) and cone angle (E) of conical cells. For the quantification of cone height, ns indicating no significant difference (Mann–Whitney U test, P > 0.05) between the data sets from *an-t1*, *an-t2* and *an-t1 COM 1#* compared with WT Col-0 (P = 0.2033, P = 0.1293, and P = 0.51330, respectively). For the quantification of cone angle, asterisks indicate a significant difference (Mann–Whitney U test, ***P < 0.001) between the data sets from *an-t1* and *an-t2* compared with WT Col-0 (P = 0.0009, P = 0.0006, respectively), and ns indicates no significant difference (Mann–Whitney U test, P > 0.05) between the data sets from *an-t1 COM 1#* compared with WT Col-0 (P = 0.7822). Values are given from more than 260 cells of 25 petals. (F and G) dihydroethidium (DHE)- and CM-H2DCFDA-stained conical cells from stage 14 flowers for O_2_^• –^ and H_2_O_2_ analysis in WT, *an-t1*, *an-t2*, and *an-t1 COM1#*. Scale bar represents 10 μm. (H and I) Quantification of DHE-detected O_2_^• –^ (H) and CM-H2DCFDA-detected H_2_O_2_ (I) in conical cells. For comparative O_2_^• –^ and H_2_O_2_ analysis, a region of interest (ROI) at the conical cells was quantified by ImageJ. For the quantification of average fluorescent intensity of DHE-detected O_2_^• –^ , asterisk indicates a significant difference (Mann–Whitney U test, *P < 0.05) between the data sets from *an-t1* and *an-t2* compared with WT Col-0 (P = 0.024, P = 0.024, respectively), and ns indicating no significant difference (Mann–Whitney U test, P > 0.05) between the data sets from *an-t1 COM 1#* compared with WT Col-0 (P = 0.6423). For the quantification of average fluorescent intensity of CM-H2DCFDA-detected H_2_O_2_, asterisk indicates a significant difference (Mann–Whitney U test, **P < 0.01) between the data sets from *an-t1* and *an-t2* compared with WT Col-0 (P = 0.0025, P = 0.0016, respectively), and no significant difference (Mann–Whitney U test, P > 0.05) between the data sets from *an-t1 COM 1#* compared with WT Col-0 (P = 0.4304). For the boxplots, the box extending from the lower to upper quartile values of the data, with a line representing the data medians. The whiskers extending past 1.5 of the interquartile range. Values are given from more than 110 cells of 15 petals.

To identify regulatory components involved in controlling conical cell expansion in the petal blade epidermis, we screened more than 500 mutant homozygous lines from the Arabidopsis Biological Resource Center (ABRC) for mutants with abnormal conical cell phenotypes [21]. From this screen, we identified one mutant line, SALK_026489C, previously named *an-t1* [34], with a T-DNA insertion causing a null allele of the gene *AN* (*AT1G01510*) (S1A and S1B Fig). The *an-t1* mutant showed reduced tip sharpening of conical cells, exhibiting a phenotype of conical cells with wider tip angles (Fig 1A–1E). As shown in Fig 1A, we quantified cone parameters (cone angles and cone heights) of conical cells from wild type with normal conical tips and mutant with swollen conical tips. Quantification data showed that conical cells of the *an-t1* mutant had increased cone angles but no alternation in cone heights compared with those of the wild type (Fig 1A–1E). To further confirm the role of AN in conical cell shaping, one additional T-DNA insertion null mutant of the *AN* gene, *an-t2* (WiscDslox329F05) (S1A and S1B Fig), was obtained from the ABRC. The *an-t2* mutant exhibited a conical cell phenotype similar to *an-t1* (Fig 1A–1E). Next, we performed the complementation experiment for *an-t1* mutant line by introducing the coding sequence of the *AN* gene fused with the GFP tag under the control of *AN*’s promoter into *an-t1*. We obtained more than 30 transgenic lines that could fully complement the previously reported *an-t1* mutant phenotypes [23,24], in terms of narrow cotyledons and leaves, less-lobed pavement cells, and reduced trichome branches. One transgenic line, *an-t1 COM #1*, was selected for phenotypic analyses (Figs 1A–1E and S1C–S1F), and was shown to express the *AN* gene fused with GFP (S1G Fig). Expression of *AN*^*pro*^::*GUS* showed strong signals in petals throughout petal developmental stages (S1H Fig). Furthermore, conical cells of the *an-t1* mutant had similar geometric shape as the wild type at petal development stages 8–10, but displayed increased tip angles at petal development stage 11 and beyond compared with the wild type (S1I–S1K Fig). Taken together, these results demonstrated that AN plays a role in promoting the tip sharpening of conical cells at late developmental stages.

Given that a previous report has shown that loss of AN function causes accumulation of high ROS levels in leaves by an unknown mechanism [34], we investigated whether the wider-angled tips of conical cells of *an* mutants attributed to this abnormal ROS accumulation. Thus, we compared ROS levels between wild type and *an* mutants. ROS contain many molecules, with superoxide radical (O_2_^• –^), the precursor for most other ROS, and hydrogen peroxide (H_2_O_2_), being the two major components [35]. We investigated O_2_^• –^ and H_2_O_2_ distribution using nitroblue tetrazolium (NBT) [36] and 3,3’-diaminobenzidine (DAB) staining [37], respectively. We found that both the *an-t1* and *an-t2* mutants accumulated higher levels of O_2_^• –^ and H_2_O_2_ in their petals compared with those of the wild type (S2A and S2B Fig). Furthermore, we examined the O_2_^• –^ and H_2_O_2_ distribution at the cellular resolution. Using the fluorescent dye dihydroethidium (DHE) and 2',7'-dichlorofluorescein diacetate (CM-H2DCFDA) [38,39] to monitor O_2_^• –^ and H_2_O_2_ production by laser scanning confocal microscopy, respectively, we found that both the *an-t1* and *an-t2* mutants’ mature conical cells had significantly higher levels of O_2_^• –^ and H_2_O_2_ compared with the wild type (Fig 1F–1I). Moreover, the *an-t1 COM #1* complementation line could fully rescue the ROS levels of *an-t1* (Fig 1F–1I). Interestingly, qRT-PCR analysis revealed that the *AN* expression was down-regulated by H_2_O_2_ treatment (S2C Fig). Consistently, western blot analysis demonstrated that AN protein was also down-regulated by H_2_O_2_ treatment (S2D–S2F Fig), implying that H_2_O_2_ negatively regulates *AN* expression. Taken together, these results suggested that AN plays a role in conical cell shaping probably by negatively regulating ROS production.

The above-mentioned results raised the possibility that ROS levels play crucial roles in normal conical cell expansion of petal adaxial epidermal cells over the course of development. Thus, we quantitatively examined the O_2_^• –^ and H_2_O_2_ distribution in developing wild-type petals. Notably, we detected low levels of O_2_^• –^ but not H_2_O_2_ in the petals at stage 8 (S3 Fig), where petal adaxial epidermal cells display a flat shape. Remarkably, the levels of O_2_^• –^ were slightly decreased in the petal adaxial epidermis at stages 9 and 10 (S3 Fig), in which epidermal cells displayed roughly hemispherical morphologies, while H_2_O_2_ levels were almost not detected. At stage 10 and beyond, both the levels of O_2_^• –^ and H_2_O_2_ became more and more apparent (S3 Fig) in the middle region of the petal blade’s adaxial epidermis, peaking at stage 14, as the petal grew and conical cells became increasingly sharpened. This may indicate that O_2_^• –^ plays crucial roles at both early and late stages during conical cell expansion and that H_2_O_2_ plays roles at later development stages. Notably, adaxial epidermal cells in top region of the petal claws displaying relatively flat but not conical shape, did not show increased ROS levels in the late phases of petal development (S4 Fig). Therefore, these results demonstrated that ROS show a developmentally regulated accumulation pattern during conical cell expansion.

We next compared ROS levels between wild type and *an* mutants at multiple developmental stages. Quantitative analysis showed that H_2_O_2_ levels in the *an-t1* and *an-t2* mutants were significantly higher at stage 10 and beyond compared with those of the wild type (S5 Fig), this being consistent with the observation that AN affected conical cell development at late stages. Furthermore, the *an-t1 COM #1* complementation line could fully rescue H_2_O_2_ levels (S5 Fig).

To investigate whether the AN-ROS pathway is a general mechanism important for cell shape in many different cell types, we next compared ROS levels in leaf pavement cells and trichomes between *an* mutants and the wild type. The *an-t1* mutant accumulated higher levels of O_2_^• –^ and H_2_O_2_ in their leaf pavement cells and trichomes compared with those of the wild type (S6 Fig). These findings suggested that AN plays a general role in regulating ROS levels in diverse cell types.

### ROS homeostasis is required for normal conical cell expansion

**Fig 2:**
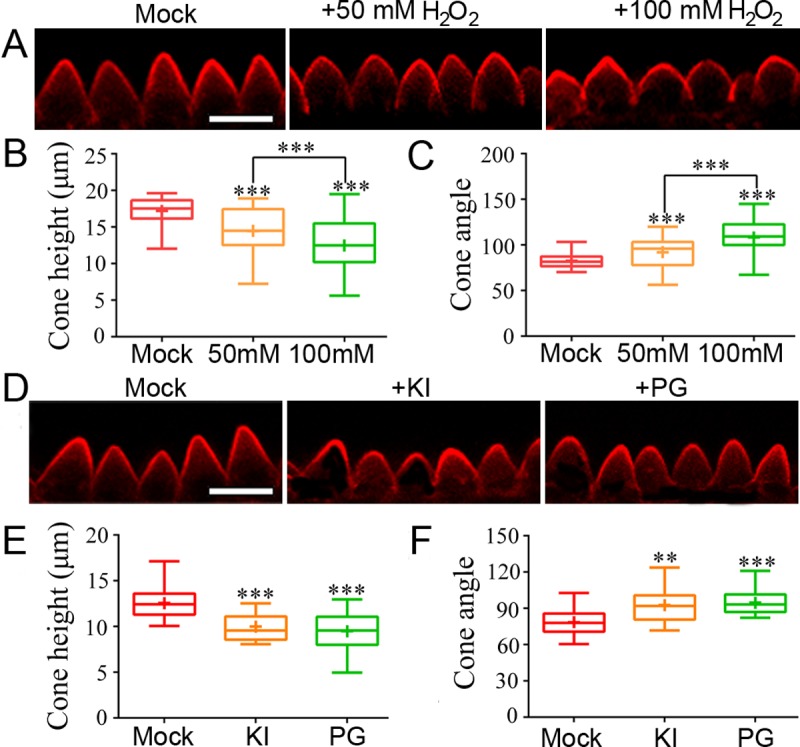
ROS homeostasis is essential for normal conical cell expansion. (A) Exogenously supplied H_2_O_2_ leading to reduced tip sharpening of conical cells. WT flower buds at stage 7 were treated by mock, 50 mM H_2_O_2_, and 100 mM H_2_O_2_, respectively. Conical cells from stage 14 mature petals were visualized by confocal. Scale bar represents 20 μm. (B and C) Boxplots of cone height (B) and cone angle (C) of conical cells. For the quantification of cone height, asterisks indicate a significant difference (Mann–Whitney U test, ***P < 0.001) (P = 0.00018, P = 0.00013, P = 0.00056, respectively). For the quantification of cone angle, asterisks indicate a significant difference (Mann–Whitney U test, ***P < 0.001) (P = 0.00043, P = 0.00019, P = 0.00035, respectively). For all data sets used for quantifications, n = 120 cells from 10 petals. (D) Reduced endogenous ROS levels causing reduced tip sharpening of conical cells. WT flower buds at development stage 7 were exposed to three different treatments: mock, 1 mM KI, and 5 mM n-propyl gallate (PG). Scale bar = 20 μm. (E and F) Quantification of cone height (E) and cone angle (F) of conical cells. For the quantification of cone height, asterisks indicate a significant difference (Mann–Whitney U test, ***P < 0.001) (P = 0.00022, P = 0.00011, respectively). For the quantification of cone angle, asterisks indicate a significant difference (Mann–Whitney U test, **P < 0.01, ***P < 0.001) (P = 0.00125, P = 0.00016, respectively). For all data sets used for quantifications, n = 120 cells of 20 petals. For the boxplots, the box extending from the lower to upper quartile values of the data, with a line representing the data medians. The whiskers extending past 1.5 of the interquartile range.

The observations that mature petals of *an* mutants displayed reduced tip sharpening of conical cells and increased ROS levels raise the hypothesis that ROS act downstream of AN to modulate conical cell expansion in petal adaxial epidermis. To test this hypothesis, we next sought to determine whether exogenously supplied ROS was sufficient to generate conical cells with wider tip angles similar to those of *an* mutants. We then used 50 mM and 100 mM H_2_O_2_ to treat flower buds of stage 7 from wild-type inflorescences, respectively. We repeated the H_2_O_2_ application four additional times, with 24 hours between each application, to generate long-term H_2_O_2_ effects. Indeed, analysis of conical cell phenotypes of mature petals after H_2_O_2_ treatment showed that, H_2_O_2_ application induced a wide-angled tip phenotype in conical cells in a H_2_O_2_ dose-dependent manner (Fig 2A–2C).

To further investigate the biological functions of ROS in conical cell shape control, we next reduced endogenous levels of O_2_^• –^ and H_2_O_2_, respectively. Wild-type flower buds of stage 7 were treated with 5 mM n-propyl gallate (PG) or 1 mM KI, respectively. To generate long-term drug effects, we repeated the treatments four additional times, with 24 hours between each application. The flower buds developed into stage 14 mature petals were used for phenotypic analyses of conical cell. We found that removal of either O_2_^• –^ or H_2_O_2_ could result in conical cells with wider tip angles (Figs 2D–2F and S7). Surprisingly, removal of H_2_O_2_ by KI treatment caused accumulation of high levels of O_2_^• –^ in mature petal epidermis (S8 Fig). This result reflected that H_2_O_2_ negatively regulates O_2_^• –^ biosynthesis during conical cell shaping through an unknown mechanism, consistent with a recent study showing that H_2_O_2_ negatively regulates O_2_^• –^ levels in *A*. *thaliana* stem cells [39]. However, the conical cell phenotypes induced by eliminating endogenous ROS levels were less severe than those by exogenous H_2_O_2_ applications. Furthermore, consistently with the observation that H_2_O_2_ contents were hardly detected at early stages (S3 Fig), treating wild-type flower buds with KI at stage 7 for one-time treatment led to no alternation of conical cell shape (S7 Fig). In contrast, treating wild-type flower buds with KI at either stage 9 or stage 11 for one-time treatment resulted in wide-angled tips of conical cells compared with the mock treatment (S7 Fig). Notably, treatment with PG at either stage 7, stage 9, or stage 11 for one-time treatment, respectively, could generate mature conical cells with wider tip angles (S7 Fig). Taken together, these results suggested that endogenous ROS is required for normal conical cell expansion.

### Endogenously increased ROS levels by Rho GTPase ROP2 overexpression suppresses tip sharpening of conical cells

**Fig 3:**
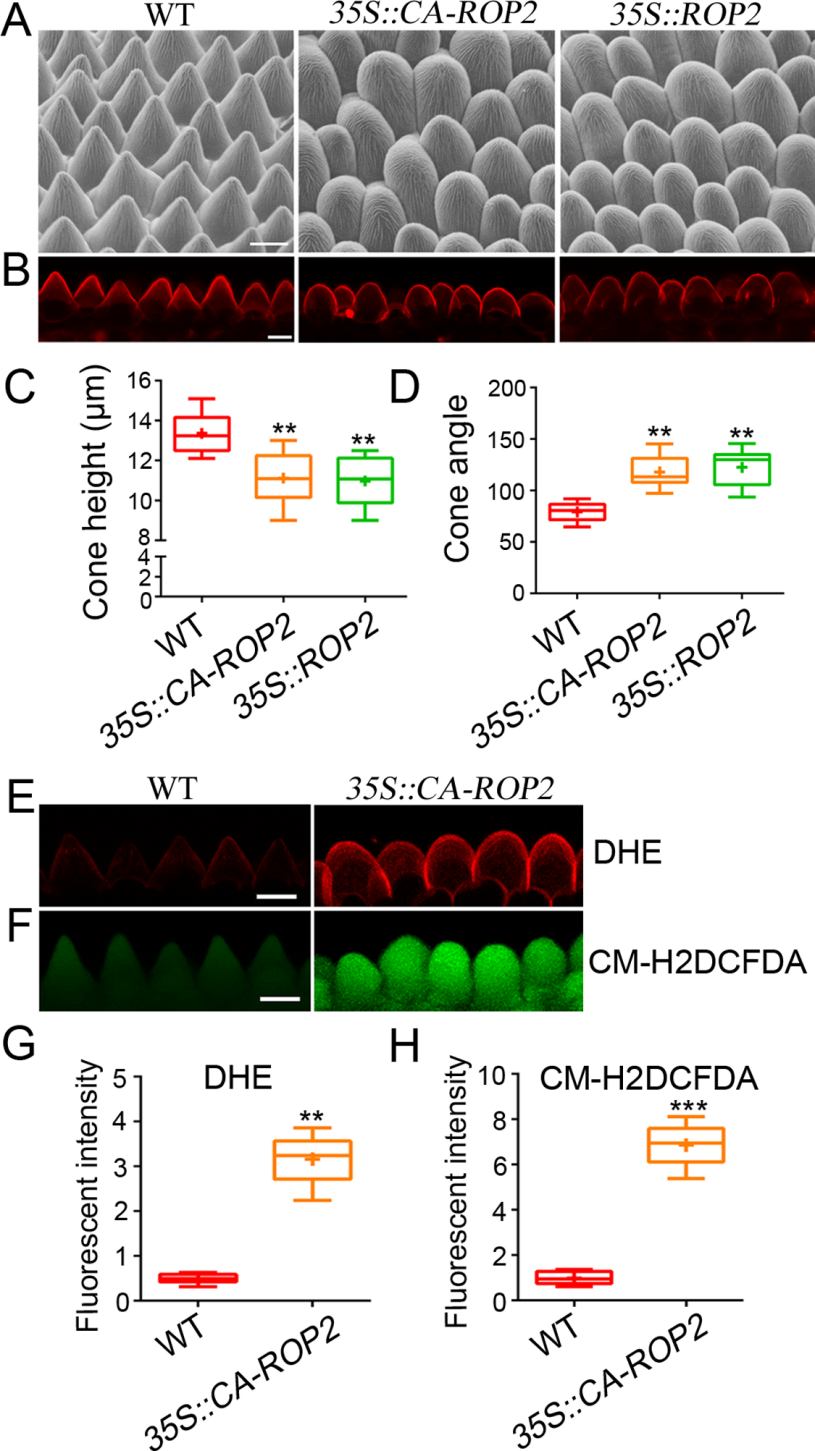
ROP2 overexpression leading to high levels of ROS and swollen conical cells. (A and B) ROP2 overexpression leads to a reduced tip sharpening phenotype in conical cells. Scanning electron microscope (A) and confocal (B) images of conical cells of WT, *35S*::*CA-ROP2*, and *35S*::*ROP2* from stage 14 flowers. Scale bars represent 10 μm. (C and D) Quantification of conical cell phenotypes from WT, *35S*::*CA-ROP2*, and *35S*::*ROP2*. For the quantification of cone height, asterisks indicate a significant difference (Mann–Whitney U test, **P < 0.01) (P = 0.0095, P = 0.0051, respectively). For the quantification of cone angle, asterisks indicate a significant difference (Mann–Whitney U test, **P < 0.01) (P = 0.00261, P = 0.00123, respectively). Values are given as the mean ± SD of more than 300 cells from 20 petals from three independent plants. (E and F) DHE- and CM-H_2_DCFDA-stained conical cells of WT and *35S*::*CA-ROP2* from stage 14 flowers. Scale bars represent 10 μm. (G and H) Quantification of DHE-detected O_2_^• –^ (G) and CM-H2DCFDA-detected H_2_O_2_ (H) in conical cells. For comparative O_2_^• –^ and H_2_O_2_ analysis, a region of interest (ROI) in the conical cells was quantified by ImageJ. For the quantification of DHE-detected O_2_^• –^, asterisks indicate a significant difference (Mann–Whitney U test, **P < 0.01) (P = 0.0033). For the quantification of CM-H2DCFDA-detected H_2_O_2_, asterisks indicate a significant difference (Mann–Whitney U test, ***P < 0.001) (P = 0.0005). Values are given as the mean ± SD of more than 100 cells from 10 petals.

To further confirm the role of ROS in modulating conical cell shape, we investigated whether endogenously increased ROS levels could also lead to the phenotype of wide-angled tips of conical cells. The plant Rho GTPase, which functions as a central molecular switch to control diverse cellular processes [40], has been shown to positively regulate endogenous ROS levels through directly binding to NADPH oxidase [41]. Thus, we investigated conical cell phenotypes in transgenic lines overexpressing *ROP2*. In agreement with the role of Rho GTPase in ROS production [41], analyses of petals in transgenic *Arabidopsis* plants overexpressing *ROP2* (*ROP2 OX*) or a constitutively active mutant *ROP2* gene (*CA-ROP2*) [42] under the control of the 35S promoter showed significantly increased ROS levels throughout the late phases of petal development (S9 Fig). In agreement with the observation that wide-angled tips of conical cells are correlated with accumulation of high levels of ROS (Fig 3A**–**3D), mature petals from both *ROP2 OX* and *CA-ROP2* plants displayed swollen conical cells, with higher levels of both O_2_^• –^ and H_2_O_2_ (Fig 3E**–**3H). However, this phenotype was more severe than the *an-t1* and *an-t2* mutants. This may reflect that ROP GTPase signaling acts through multiple downstream components [40].

### Identification of potential AN interactors

Given that both *an* mutants and *CA-ROP2* plants exhibited a similar conical cell phenotype, correlating with accumulation of high ROS levels, we therefore explored the relationship between AN and ROP2. Thus, we tested whether AN functions to affect ROS production by either directly or indirectly activating ROP2. We then examined ROP2 activity in the inflorescences of wild type and *an* mutant lines. A comparison of the activation status of ROP2 in the wild type and the mutants *an-t1* and *an-t2* showed no significant differences (S10 Fig), whereas the *spk1-4* mutant with a mutation in *SPIKE1* as a control had reduced ROP2 activity as described previously (S10 Fig) [43]. This result suggested that AN may not act upstream of ROP2 in regulating ROS production.

To investigate the molecular mechanism by which AN regulates ROS levels, we identified potential interacting proteins of GFP-AN using a GFP-trap-immunoprecipitation based approach coupled with mass spectrometry (MS)-based protein identification. Proteins were isolated from whole-inflorescence tissues, excluding mature flowers and developing siliques, of a transgenic line expressing AN-GFP under the 35S promoter to pull-down GFP-AN protein complexes using GFP-binding agarose beads. Protein samples pulled-down from a transgenic line expressing GFP alone under the 35S promoter were used as a control. Co-immunoprecipitation (Co-IP)-MS analyses were conducted from three independent harvests of inflorescence tissues from both *p35S*:: *GFP-AN* line and *p35S*::*GFP* line to compose a biological triplicate. Gel analysis of the pull-down samples demonstrated a high specificity and efficiency for GFP-AN protein enrichment (S11 Fig). Pulled-down protein samples were analyzed by MS, and stringent data analyses identified proteins that are exclusive in all three GFP-AN replicates but not present in any of the control pull-downs. To identify proteins present in GFP-AN complexes that can be relevant to a role in ROS production, we chose targets already annotated with gene ontology (GO) biological processes related to oxidation-reduction process and oxidoreductase. Notably, among the identified AN-interacting proteins (S1 Table), we obtained 49 proteins directly responsible for ROS homeostasis (S1 Table). These ROS-related proteins include CATALASE 2 (CAT2) and CATALASE 3 (CAT3), which can catalyzes the breakdown of H_2_O_2_ [44]. In addition, several NAD(P) superfamily proteins involved in the oxidation-reduction process were identified as putative AN interactors (S1 Table). To confirm the role of CAT2 in regulating conical cell shape, we identified one mutant line *cat2-1* (SALK_076998) from the ABRC stock center, with a T-DNA insertion causing a null allele of the gene *CAT2* (*AT4G35090*) (S12A and S12B Fig). The *cat2-1* mutant exhibited a phenotype of conical cells with wider tip angles compared with the wild type (S12C–S12F Fig). We next sought to investigate whether AN and CAT2/CAT3 could physically interact. We used co-IP experiments to detect their interactions *in vivo*. We generated transgenic lines expressing *CAT2-GFP* and *CAT3-GFP*, respectively, and used GFP-Trap agarose beads to pull-down protein samples for the co-IP analyses. However, in our experimental conditions, the western-blotting results showed that AN may not physically interact with CAT2 and CAT3 in planta (S13 Fig). Although the direct interactions between AN and ROS-related proteins need to be further determined, the list of potential AN interactors may suggest a regulation of ROS production by AN.

### Mutations in *KTN1* result in enhanced conical cell phenotypes of *an-t1*

**Fig 4:**
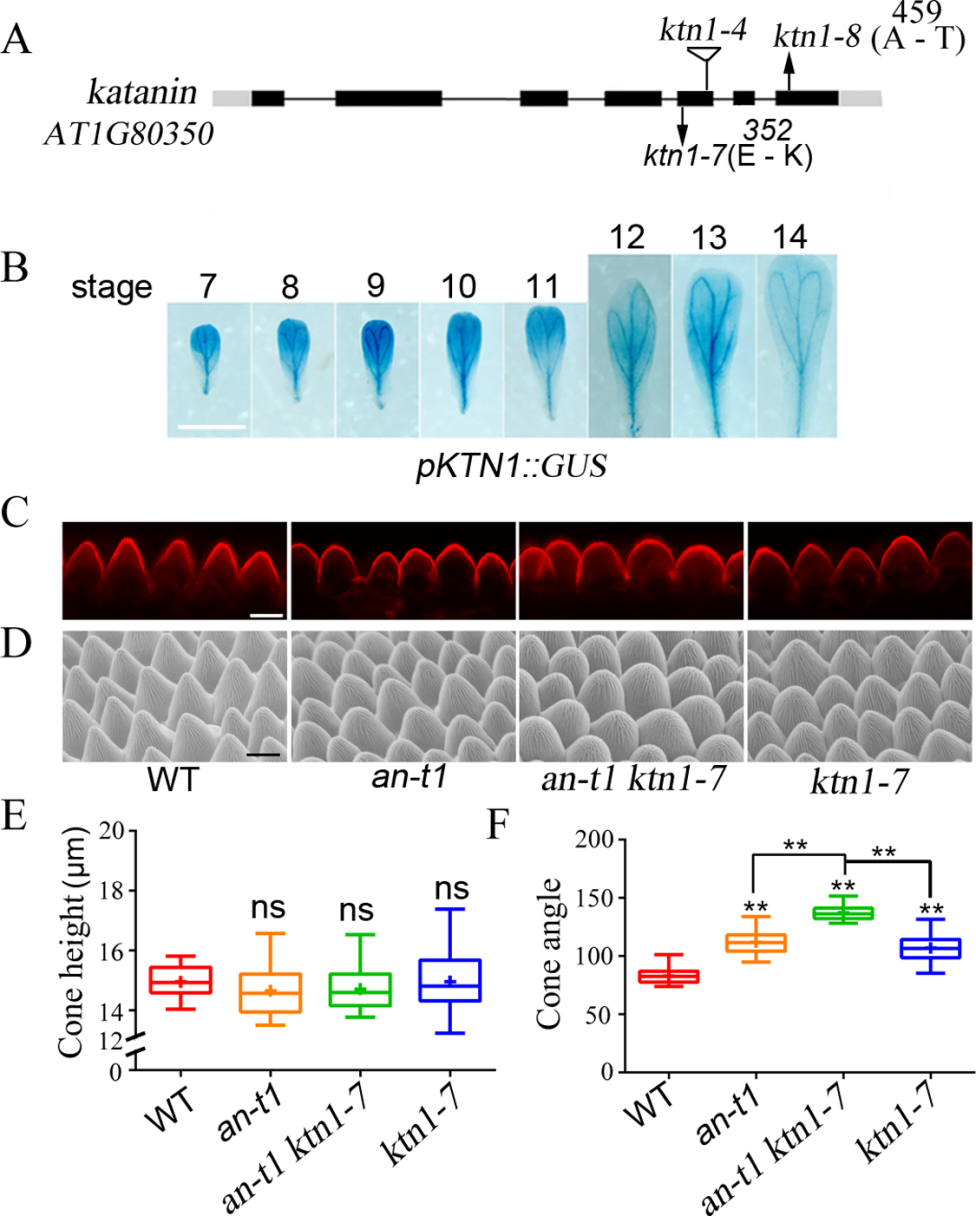
Mutations in *KTN1* leading to enhanced conical cell phenotypes of *an-t1*. (A) Schematic representation of the *KTN1* gene, showing the nature and position of the *ktn1* mutant alleles. Arrows indicate nucleotide substitutions, and the triangle indicates T-DNA insertion. (B) *KTN1* expression activity throughout petal development stages 7 to 14 was monitored by *pKTN1*:*GUS* transgene expression. Scale bar = 1 mm. (C and D) Confocal (C) and scanning electron microscope (D) images of conical cells of WT, *an-t1*, *an-t1 ktn1-7*, and *ktn1-7* from stage 14 mature petals. Scale bars represent 10 μm. (E and F) Quantification of cone height (E) and cone angle (F) of conical cell phenotypes. For the quantification of cone height, ns indicates no significant difference (Mann–Whitney U test, P > 0.05) between the data sets compared with WT Col-0 (P = 0.2633, P = 0.11099, P = 0.31530, respectively). For the quantification of cone angle, asterisks indicate a significant difference (Mann–Whitney U test, **P < 0.01) (for *an-t1* vs WT, P = 0.00359; for *an-t1 ktn1-7* vs WT, P = 0.00129; for *ktn1-7* vs WT, P = 0.0017; for *an-t1 ktn1-7* vs *an-t1*, P = 0.00223; for *an-t1 ktn1-7* vs *ktn1-7*, P = 0.0022). Values are given as the mean ± SD of more than 300 cells from 20 petals.

To identify genetic components that function together with AN in controlling conical cell expansion, we mutagenized *an-t1* seeds with ethyl methanesulfonate (EMS) and conducted a genetic screen for mutants with enhanced conical cell expansion defects of *an-t1*. From this screen, we identified two enhancer mutants. Backcrosses to *an-t1* and subsequent genetic analyses revealed that these two mutants were allelic and both harbored a recessive mutation. Sequencing of the *KTN1* genomic DNA in these two mutants revealed a G-to-A mutation, resulting in amino acid alterations in the conserved ATPase domain of the *KTN1* genes (Fig 4A). Thus, these two mutants were named *ktn1-7*and *ktn1-8*, respectively. Notably, these mutants displayed short roots, compact rosette leaves, and dwarf plants (S14A–S14C Fig), reminiscent of the *ktn1-4* mutant phenotypes [45]. The *ktn1-8* allele had a weaker phenotype compared with those of the *ktn1-4* allele (S14A–S14C Fig), suggesting that it is a novel weak allele. A transgenic line expressing a *KTN1*^*pro*^::*GUS* construct showed strong GUS signals in petals throughout petal development stages (Fig 4B), consistent with the role of KTN1 in petal conical cell shaping.

Conical cells of the *ktn1-7* and *ktn1-8* single mutants had wider tip angles than the wild type, but displayed similar phenotypes to *an-t1* (Figs 4C–4F and S14D–S14G). Notably, both the *an-t1 ktn1-7* and *an-t1 ktn1-8* mutants had dramatically enhanced cell expansion defects, exhibiting extremely swollen petal adaxial epidermal cells, with a hemispherical shape instead of a conical shape, compared with the wild type and even the *an-t1* mutant (Figs 4C–4F and S14D–S14G). Taken together, these findings suggest that AN and KTN1 have a genetic interaction, and may act in parallel and functionally related processes during epidermal cell shaping.

We next sought to investigate whether AN and KTN1 could physically interact. We used co-immunoprecipitation experiments to detect their interactions *in vivo* by transiently coexpressing *35S*::*GFP-AN* with *35S*::*KTN1-My*c in *Nicotiana benthamiana* leaves. Transiently coexpressed *35S*::*GFP* and *35S*::*KTN1-Myc* were used as negative controls. In our experimental conditions, the result showed that KTN1-Myc was not detected in either the immunoprecipitated AN-GFP complex or the GFP complex (S14H Fig), indicating that AN may not physically interact with KTN1 in planta. Consistently, we did not detect the KTN1 protein from our Co-IP MS analyses in the *AN-GFP* line.

### The AN-ROS pathway cooperates with KTN1 to organize microtubule orientation during conical cell expansion

**Fig 5:**
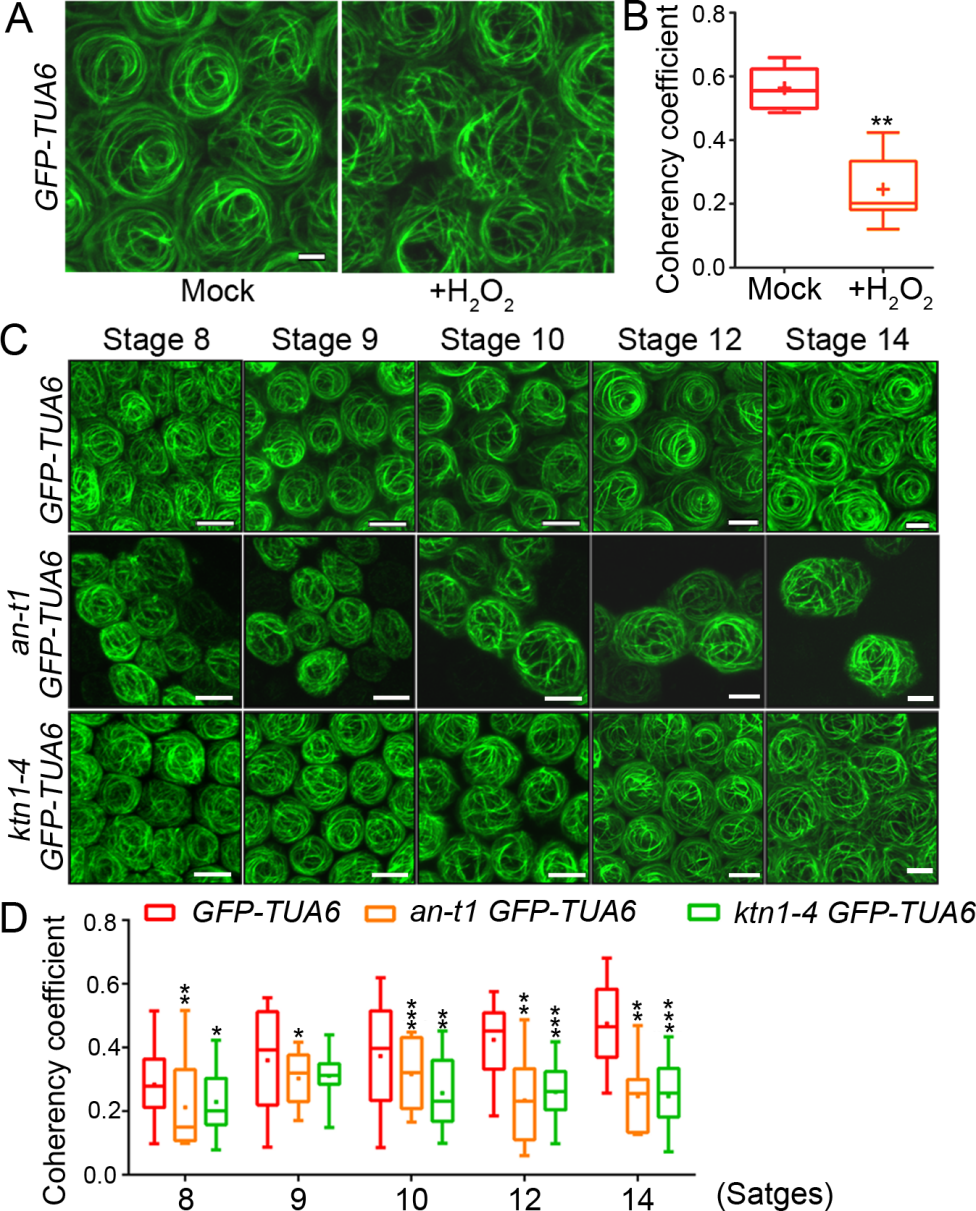
ROS-dependent microtubule orientation during conical cell shaping. (A) Exogenously supplied 100 mM H_2_O_2_ suppressed microtubule orientation into well-ordered circumferential arrays. Scale bar represents 5 μm. Representative confocal images were generated by top-down 2D maximum projections of Z-stacks. (B) Quantification of microtubule alignment. The microtubule alignment measurement was carried out with "OrientationJ", a ImageJ plug-in, to calculate the directional coherency coefficient of the fibers. A coherency coefficient close to 1 represents a strongly coherent orientation of the microtubules. Asterisks indicate a significant difference (Mann–Whitney U test, **P < 0.01) (P = 0.00233). Values are given from more than 70 cells of 15 petals. (C) Visualization of microtubules in conical cells of wild type, *an-t1*, and the *ktn1-4* mutant at the indicated developmental stages. Representative confocal images were generated by top down 2D maximum projections of Z-stacks. Scale bars represent 5 μm. (D) Quantification of microtubule alignment in conical cells. Asterisks indicate a significant difference (Mann–Whitney U test, *P < 0.05, **P < 0.01, ***P < 0.001) (from left to right, P = 0.0071, P = 0.03454, P = 0.02066, P = 0.05546, P = 0.00063, P = 0.00422, P = 0.00307, P = 0.00054, P = 0.00105, P = 0.00057). For all genotypes, n = 80 cells from 15 petals).

Recent reports have suggested that ROS influence cytoskeleton dynamics [46], and that H_2_O_2_ directly activates the MAPK cascade to modulate the activities of MAP65 proteins, consequently affecting microtubule orientation [46–49], although the underlying molecular mechanisms remain to be further explored. We next tested whether ROS are essential for cortical microtubule organization during conical cell shaping. Firstly, we investigated whether exogenously supplied H_2_O_2_ could lead to alterations in microtubule orientation using a microtubule reporter line expressing GFP-tagged α-tubulin 6 (GFP-TUA6) [50]. We used 100 mM H_2_O_2_ to treat flower buds of stage 7 from inflorescences of the *GFP-TUA6* reporter line, and the same treatment was repeated four additional times, with 24 hours between each application, to generate long-term H_2_O_2_ effects. After treatments, we visualized microtubule arrays from the top view of the non-folded mature petals in confocal Z sections, allowing for the top view of conical cells. We quantified the microtubule alignment with "OrientationJ", a ImageJ plug-in, used for calculating the directional coherency coefficient of the fibers [51]. A coherency coefficient close to "1" represents a strongly coherent orientation of the microtubules. We found that adaxial epidermal cells without H_2_O_2_ treatments had well-ordered circumferential microtubule arrays aligned perpendicular to the axis of conical outgrowth (Fig 5A and 5B), consistently with our previous report [21]. In contrast, adaxial epidermal cells of mature petals with H_2_O_2_ treatments displayed randomly oriented microtubules with reduced coherency (Fig 5A and 5B). This result suggested that high levels of ROS accumulation inhibited microtubule ordering during conical cell shaping. Consistently with these findings, we observed randomly oriented microtubules in the mature conical cells of the *CA-ROP2* line (S15A and S15B Fig), which was shown to accumulate higher ROS levels. We next investigated the effects of eliminating endogenous ROS on microtubule organization. Notably, we found that eliminating either O_2_^• –^ or H_2_O_2_ led to mature conical cells with disordered microtubule arrays with reduced coherency (S15C and S15D Fig). Thus, these results suggested that ROS homeostasis mediated microtubule orientation into well-ordered circumferential arrays in petal conical cells, although the underlying mechanism remains to be further explored.

To test whether ROS play a general role in regulating cell morphogenesis and microtubule organization in different cell types, we next investigated the effects on microtubule arrangements in both cotyledon pavement cells and leaf trichomes after exogenous H_2_O_2_ application. Notably, analysis of cotyledon pavement cell phenotypes after H_2_O_2_ treatment showed that, H_2_O_2_ application induced a phenotype of reduced lobe length in pavement cells, showing severe defects of interdigitated growth in a H_2_O_2_ dose-dependent manner (S16A–S16D Fig). It has been reported that microtubule arrays play crucial roles in leaf pavement cell shaping [52,53]. Consistently, H_2_O_2_ application induced increased alignment of microtubules in pavement cells in a H_2_O_2_ dose-dependent manner (S16E and S16F Fig). Exogenous H_2_O_2_ treatment leaded to generate smaller leaves with shorter trichomes but no alternation of trichome branching (S17A–S17E Fig). Furthermore, H_2_O_2_ treatment resulted in increased alignment of microtubules in trichomes (S17F and S17G Fig), which was similar to the result observed in leaf pavement cells. The observations that exogenous H_2_O_2_ treatment resulted in disordered microtubule arrays in petal conical cells and increased microtubule ordering in both pavement cells and trichomes, respectively, suggested that ROS may play diverse roles in modulating microtubule organizations in different cell types, although the underlying molecular mechanisms need to be further investigated.

Based on the above-mentioned results showing that ROS played a role in mediating microtubule ordering, AN inhibited ROS production during conical cell development, and that AN and KTN1 acted in parallel pathways to modulate conical cell expansion, we hypothesized that AN acts through ROS to modulate microtubule orientation and that the AN-ROS and KTN1 pathways converge at a node to affect microtubule ordering during conical cell expansion. To test this hypothesis, we first compared microtubule organization patterns of the *an-t1* mutant with the wild type. Microtubule arrays in wild-type cells became increasingly ordered over the course of conical cell development and displayed well-ordered circumferential arrays at stage 14 of petal development (Fig 5C–5D), consistently with our previous report [21]. In contrast, *an-t1* mutant conical cells that were shown to accumulate high ROS levels had randomly oriented microtubule arrays with reduced coherency throughout early and late developmental stages (Fig 5C–5D), consistently with the observation that high levels of ROS accumulation caused wide-angled tips of conical cells with disordered microtubule arrays. Furthermore, in agreement with the previous report [21], the *ktn1-4* mutant conical cells had randomly oriented microtubule arrays, similar to those observed in the *an-t1* conical cells (Fig 5C–5D). Despite the conical cells of the *an-t1 ktn1-4* double mutant displayed more severe defects than the single mutants, the *an-t1 ktn1-4* double mutant conical cells displayed randomly oriented microtubule arrays, similar to those observed in the *an-t1* or the *ktn1-4* single mutant (S18 Fig).

Given that it is well established that KTN1 directly affects microtubule ordering through its severing activity at both microtubule nucleation and crossover sites [54,55], KTN1 may function in microtubule orientation independently of ROS. As predicted, analysis of ROS accumulation in mature petals of the *ktn1-4* mutant showed no significant differences as compared to the wild type (S19 Fig). Taken together, these results suggest that the AN-ROS pathway and KTN1 acted in parallel to modulate microtubule organization and conical cell shaping.

## Discussion

ROS function as signaling molecules for organ growth and normal cellular processes such as cell growth and cell division and differentiation [26–29]. Our results provide definitive evidence for a role of ROS in modulating conical cell expansion of petal adaxial epidermal cells.

Previous reports showed that AN promotes pavement cell interdigitation in leaves, correlating with negatively regulating cortical microtubule ordering [23,24], although the detailed molecular mechanism is unclear. Our findings in this study showed that AN promotes tip sharpening of conical cells in petals, correlating with positively regulating microtubule ordering, and that AN negatively regulates ROS levels, which in turn affects microtubule organization. Notably, we demonstrated that AN also plays a negative role in regulation of ROS levels in leaf pavement cells and trichomes. Therefore, we proposed that the AN-ROS-microtubule pathway is a general mechanism important for cell shaping in many different cell types.

In contrast to the role of AN in negatively regulating ROS production, ROP2 plays a role in positively regulating ROS production during conical cell shaping. AN and ROP2 may act antagonistically to regulate ROS homeostasis, although the molecular mechanisms by which AN suppresses and ROP2 promotes ROS production, respectively, remain to be further explored. Therefore, we propose that an endogenous balance between ROS accumulation and removal must be achieved and tightly regulated to generate microtubule reorientation and normal conical cell expansion of petal adaxial epidermal cells. Notably, loss of KTN1 function does not result in alternation in ROS levels in conical cells, suggesting that KTN1-mediated microtubule re-orientation may act in parallel with ROS signals during conical cell tip sharpening.

Emerging evidence suggests that ROS and redox cues have effects on microtubule behaviors [46–49]. Consistently with these reports, we demonstrated that both H_2_O_2_ treatment and endogenously increased ROS levels induced by either loss of AN function or *ROP2* overexpression resulted in reduced microtubule ordering of the conical cell. Furthermore, the effects of diverse ROS types on cell wall properties have been well studied [46]. ROS play critical roles in both cell-wall stiffening and loosening by promoting the formation of crosslinks between cell wall polysaccharides and glycoproteins, or by cleaving cell wall polysaccharides, respectively. Therefore, based on these findings, we cannot rule out the possibility that ROS also play a role in conical cell shaping by directly influencing cell wall properties. How ROS-mediated signaling regulates microtubule orientation remains unclear and will require extensive research in the future.

Given that previous reports have shown that KTN1-mediated microtubule severing plays critical roles in promoting microtubule rearrangements in response to mechanical stress in both the *A*. *thaliana* shoot apical meristem and cotyledon pavement cells [56,57], it is possible that mechanical forces could generate a signal to induce microtubule orientation in a KTN1-dependent manner during conical anisotropic expansion of petal adaxial epidermal cells. Interestingly, cells can respond to mechanical signals generated by cell geometry, providing a pervasive feedback on growth [58]. Future studies should aim to uncover the role of mechanical forces during conical cell shaping.

**Fig 6:**
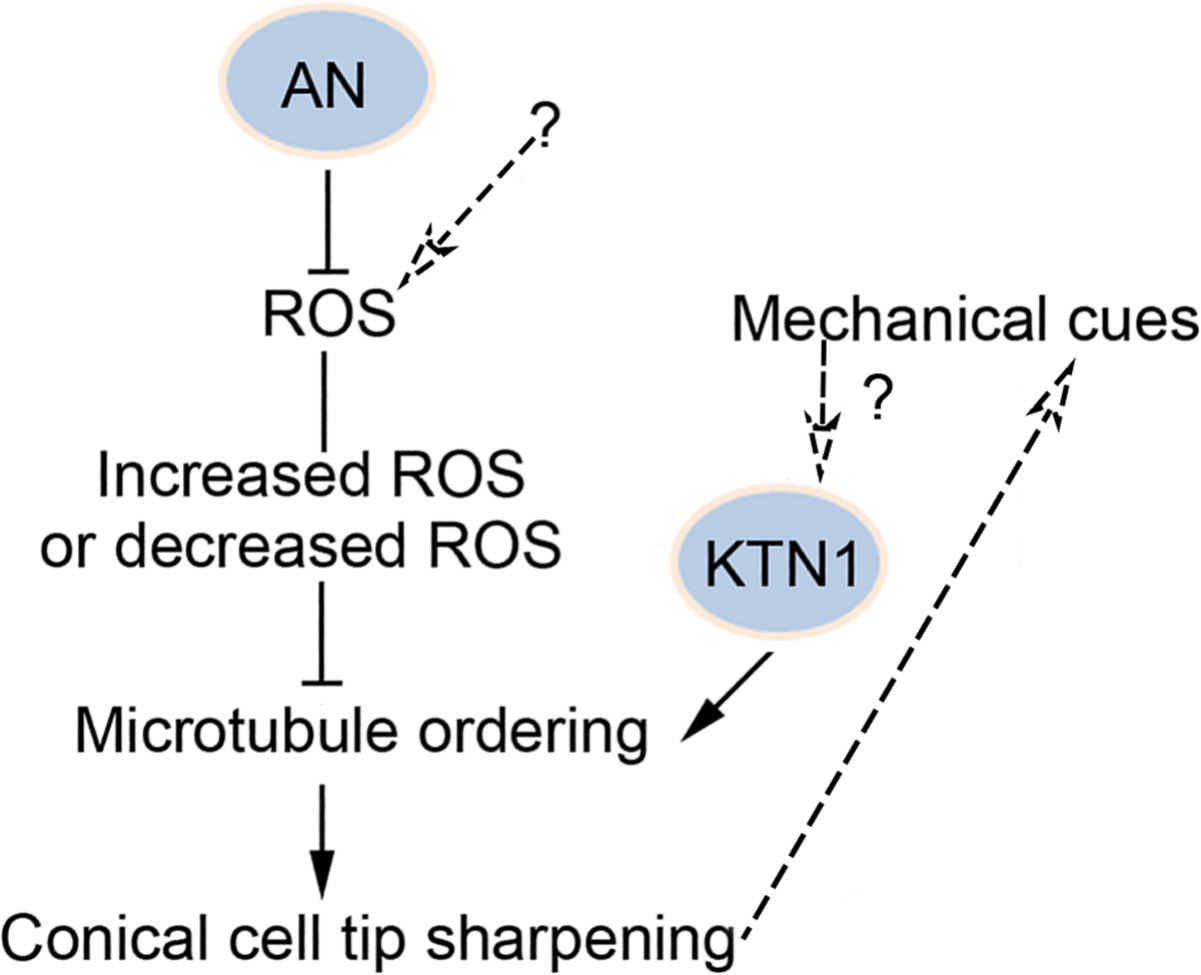
Proposed model of ROS-dependent regulation of microtubule orientation and conical cell tip sharpening. A model depicting the molecular basis of ROS-dependent microtubule orientation and conical cell tip sharpening. We propose that AN and KTN1 jointly function to modulate microtubule orientation and conical cell shaping. AN functions to suppress ROS production, respectively. Both increased and decreased ROS suppress microtubule orientation into well-ordered circumferential arrays. We hypothesized that mechanical forces could generate a signal to induce microtubule orientation, and that conical cells could respond to mechanical cues generated by cell geometry, providing a feedback loop.

On the basis of our findings, we proposed a model to explain the molecular mechanism underlying ROS-dependent microtubule orientation in the regulation of conical cell shaping (Fig 6). According to this model, the AN-ROS pathway cooperates with KTN1 to jointly reorient microtubules from random to well-ordered transverse arrays, which is critical for the tip sharpening of conical cells. We hypothesized that the re-orientation of microtubules from random to well-ordered arrays may orient the deposition of cellulose microfilaments and generate the cell wall reinforcement throughout conical cell development [12–19], consequently maintaining conical cell expansion and forming the final characteristic cell shape. Also, we hypothesized that mechanical forces could generate a signal to induce microtubule orientation. Conical cells could respond to mechanical cues generated by cell geometry, providing a feedback loop to define the final cell shape.

## Materials and methods

### Plant materials and growth conditions

All *A*. *thaliana* seeds used in this study were of the Columbia-0 (Col-0) ecotype. The *ktn1-4* (SAIL_343_D12), *an-t1* (SALK_026489C), and *an-t2* (CS851381) were obtained from the Arabidopsis Biological Resource Centre. The seeds were sown in petri dishes on Murashige and Skoog medium agar supplemented with 1% (w/v) sucrose. Plants were grown in a growth room at 22°C under 16-hr light/8-hr dark cycles.

### Gene constructs and generation of transgenic plants

The sequences of primers used in this study are listed in S2 Table. The full length coding sequences (CDS) of the *AN* gene was amplified and cloned into PH35S-GFP-GW to construct *p35S*::*GFP-AN*. For Co-IP-LC-MS/MS analysis, the Ti plasmid expressing recombinant GFP-AN protein was introduced into *rdr6-11* plants [59], which suppresses gene silencing. For the complementation experiment, the *AN* promoter was amplified from wild-type genomic DNA, and was inserted at Hind III and Xba I sites of vector *p35S*::*GFP-AN* to replace the 35S promoter, generating *pAN*::*GFP-AN*. For GUS activity assays, the promoter of *AN* was amplified and cloned into *pCAMBIA1301*. For co-IP assay, *KTN1* CDS was cloned into *pGWB517* to generate *35S*::*KTN1-Myc*. *CAT2* and *CAT3* CDS was cloned into *pGWB505* to generate *35S*::*CAT2-GFP* and *35S*::*CAT3-GFP*, respectively. The Ti plasmid expressing recombinant proteins were introduced into *rdr6-11* to generate stable transgenic lines.

### Detection of ROS

Nitroblue tetrazolium (NBT) and dihydroethidium (DHE) were used for superoxide (O_2_^• –^) staining. 3,3’-diaminobenzidine (DAB) and CM-H2DCFDA were used for hydrogen peroxide (H_2_O_2_) staining.

### ROS-related treatments

For H_2_O_2_ treatments, the concentrations used were 50 mM or 100 mM H_2_O_2_. For ROS scavenging treatments, O_2_^• –^ was eliminated with 5 mM PG, and H_2_O_2_ eliminated with with 1 mM KI.

### Confocal microscopy

For confocal imaging of conical cells from the side, petal blades were folded back, allowing for the side view of conical cells, and stained with a solution containing 10 μg/ml propidium iodide for more than 10 min. Petal samples were imaged with a Zeiss LSM 880 confocal laser scanning microscope (excitation at 514 nm, emission 550-700nm). For live-confocal imaging of cortical microtubules, non-folded petals stably expressing *GFP-TUA6* were imaged with a Zeiss LSM 880 confocal laser scanning microscope (excitation at 488, emission 500-570nm). Serial optical sections were taken at 0.5-μm increments with a 40 × water or 63 × oil lens, and then were projected on a plane (i.e. maximum intensity) using Zeiss LSM 880 software.

For CM-H2DCFDA staining, petal samples were incubated in 50 mM phosphatic buffer solution (PBS, pH 7.4) containing 10 μM CM-H2DCFDA (Invitrogen, C6827) for 30 min, and then the samples were washed for three times with PBS, and observed with the Zeiss LSM 880 microscope (excitation 488 nm, emission 500–570 nm) or the Zeiss observer A1 inverted microscope. For Dihydroethidium (DHE) staining, petal samples were incubated into 50 mM PBS (pH 7.4) buffer solution containing 40 μM DHE (Sigma, D7008) for 30min, and then visualized with the Zeiss LSM 880 microscope (excitation 514, emission 520–600 nm) or the Zeiss observer A1 inverted microscope.

### Scanning electron microscopy

Petals from flower development stage 14 were dissected and directly observed with a TM-3030 table-top scanning electron microscope (Hitachi).

### Enhancer mutant screening of conical cell phenotype of *an-t1*

Approximately 10,000 seeds of *an-t1* were mutagenized using ethyl methane sulfonate. M_2_ seeds were harvested from self-fertilized M_1_ plants individually, and M_2_ lines were screened for enhanced *an-t1* petal conical cells phenotypes. Among 2,000 independent EMS-mutagenized *an-t1* lines, two candidate enhancers were identified and described in this study. Candidate mutants were backcrossed to *an-t1* three times before further phenotypic analyses.

### ROP2 activity assay

About 0.1 g of WT and mutant inflorescences were collected and frozen in liquid nitrogen, respectively. Total proteins were extracted using extraction buffer (25 mM HEPES, pH 7.4, 10 mM MgCl_2_, 10 mM KCl, 5 mM dithiothreitol, 5 mM Na_3_VO_4_, 5 mM NaF,1 mM phenylmethylsulfonyl fluoride, 1% Triton X-100, and protease inhibitor cocktail). Twenty micrograms of MBP-RIC1-conjugated agarose beads were added to the protein extracts and incubated at 4°Cfor 2h on a rocker. The beads were washed four times in wash buffer (25 mM HEPES, pH 7.4, 1 mM EDTA, 5 mM MgCl_2_,1 mM dithiothreitol, and 0.5% Triton X-100) at 4°C. GTP-bound ROP proteins associated with the MBP-RIC1 beads were boiled and used for analysis by western blot with a ROP2 antibody that was generated against the peptides QFFIDHPGAVPITTNQG (Abicode, China).

### LC-MS/MS

*A*. *thaliana s*eedlings expressing GFP-AN (in *rdr6-11* background) and, as control, GFP (35S promoter) were grown under continuous light in MS medium. Two grams 5-day-old seedlings were collected and ground in liquid N_2_, and total proteins were extracted with the buffer (25 mM Hepes-KOH, pH7.4, 10 mM MgCl_2_, 100 mM NaCl, 5mM NaF, 15% glycerol, 1% Triton X-100, proteinase inhibitor cocktail). After centrifuging at 14,000g for 15 min at 4°C, the supernatant was mixed with GFP-Trap agarose beads (gta-100, Chromotek) and rotated at 4°C for 4 hour. The immunoprecipitates were then separated in SDS-PAGE and digested with 0.025 mg/mL trypsin. The samples were put into a Thermo Scientific EASY trap column (100 μM × 2 cm, 5 μM, 100 Å, C18) for separation, and analyzed with Obitrap Fusion mass spectrometer (Thermo Finnigan, San Jose, CA). Each sample was analyzed for 60 min with a resolution of 120,000, the scanning range of 375-1500m/z, AGC target of 4e5 and injection time of 50 ms. Simultaneously, the MS2 scanning was performed with the following parameter: resolution (50,000), activation type (HCD), injection time (105ms), AGC target (1e5). The raw data operated with Proteome Discoverer 2.1 (Thermo Scientific) were searched against with protein database (TAIR10_pep_20101214.fasta), and processed with FDR (false discovery rate)≤0.01 at both the peptide and protein level.

### Co-immunoprecipitation assay

For the co-immunoprecipitation assay of AN and KTN1, The Ti plasmids *35S*::*Myc-KTN1* and *35S*::*GFP-AN*, or *35S*::*Myc-KTN1* and *35S*:: *GFP* were transiently co-expressed in *Nicotiana benthamiana* leaves, respectively. Leaf tissue was ground in liquid N_2_, and total proteins were extracted with co-IP buffer (25mM Hepes-KOH, pH7.4, 10mM MgCl_2_, 100mM NaCl, 5mM NaF, 15% glycerol, 1% Triton X-100, proteinase inhibitor cocktail). After centrifuging at 14,000g for 10 min at 4°C, the supernatant was mixed with GFP-Trap agarose beads (gta-100, Chromotek) and rotated at 4°C for 2 hour. Beads were then washed five times with ice-cold co-IP buffer (without protease inhibitors). The bound proteins were eluted from the beads with 2×SDS-PAGE sample buffer by heating at 100°C for 5 min, and analyzed by immunoblot. The primary antibody used was: anti-Myc (1:3,000, 05–724, Millipore).

For the co-immunoprecipitation assays of AN and CAT2/CAT3, *A*. *thaliana* inflorescences expressing CAT2-GFP or CAT3-GFP (in *rdr6-11* background) and, as control, GFP (35S promoter) were used for protein extraction with the buffer (25 mM Hepes-KOH, pH7.4, 10 mM MgCl_2_, 100 mM NaCl, 5mM NaF, 15% glycerol, 1% Triton X-100, proteinase inhibitor cocktail). After centrifuging at 14,000g for 15 min at 4°C, the supernatants were mixed with GFP-Trap agarose beads (gta-100, Chromotek) and rotated at 4°C for 4 hour. The bound proteins were eluted from the beads with 2×SDS-PAGE sample buffer by heating at 100°C for 5 min, and analyzed by immunoblot. The primary antibody used was anti-AN (1:2,000). For the generation of Anti-AN antibody, the amino acid sequence from 280 to 490 a.a. of AN was amplified and cloned into expression plasmid pGEX-4T (GST tag), then transformed the vector into *Escherichia coli* for protein expression. The purified protein was injected into rabbits. Then, Antiserum was extracted and purified.

### Statistical analysis

Statistical analyses were performed using Mann–Whitney U test. Not significant difference P *>* 0.05; significant difference *P *<* 0.05, **P < 0.01, and ***P *<* 0.001.

### Accession numbers

Sequence data from this article can be found in the Arabidopsis Genome Initiative or GenBank/EMBL databases under the following accession numbers: *AT1G80350* (*KTN1*), *AT1G01510* (*AN*), *AT1G20090* (*ROP2*), *AT4G35090* (*CAT2*), and AT1G20620 (*CAT3*).

## Supporting information

S1 Fig Phenotypic analyses of *AN* loss-of-function mutants. (A) Schematic representation of the *AN* gene, showing the nature and position of the *an-t1* and *an-t2* mutant alleles. Triangles indicate T-DNA insertions. (B) RT-PCR analysis of *an-t1*, *an-t2*, and *an-t1 COM #1*. (C–F) Phenotypic analyses of *an-t1*, *an-t2*, and *an-t1 COM#1*. The panels show phenotypes of 5-day-old seedlings (C), cotyledon pavement cells (D), leaf trichomes (E), and 3-week-old plants (F). Scale bars: 1 mm in *C* and *E*, 50 μm in *D*, and 1cm in *F*. (G) Western blotting analysis of protein expression of the *pAN*::*AN-GFP*/*an-t1* and *35S*::*GFP*. (H) Analysis of *AN* promoter activity throughout petal development as monitored in the promoter *AN*::*GUS* line. (I) Comparison of conical cells' geometry of wild type and the *an-t1* mutant at developmental stages 8–14. Scale bar = 20 μm. (J and K) Quantitative analyses of cone height and angle of conical cells from development stages 8 to 14 in WT and *an-t1*. Quantification of cone height (J) showed that there were no significant differences between WT and *an-t1* at each indicated developmental stages (Mann–Whitney U test, P = 0.41635, P = 0.26494, P = 0.63984, P = 0.12968, P = 0.11702, P = 0.17235, P = 0.14463). Quantification of cone height (K) showed that, at development stages 8 to 10, *an-t1* conical cells’ cone angles were similar to WT at stages 8–10 (Mann–Whitney U test, P = 0.22442, P = 0.19294, P = 0.18819) whereas at development stage 11 and beyond, *an-t1* displayed significantly increased cone angles compared with WT (***P < 0.001, Mann–Whitney U test) (from left to right, P = 0.00009, P = 0.00006, P = 0.00033, P = 0.00006). Values are given as mean ± SD of more than 180 cells of 10 petals from independent plants.
(TIF)

S2 Fig Analyses of ROS accumulation in WT and *an* mutants. (A) NBT staining for superoxide in WT and *an-t1* inflorescences and stage 14 flowers. *an-t1* had higher levels of superoxide than WT. Scale bars = 1 mm. (B) DAB staining for H_2_O_2_ in WT and *an-t1* inflorescences and stage 14 flowers. *an-t1* had higher levels of H_2_O_2_ than WT. Scale bars = 1 mm. (C) *AN* mRNA levels were decreased after H_2_O_2_ treatment. 6-day seedlings of Col-0 were treated with 100 mM H_2_O_2_ for 0h (mock), 1h, 2h, and 3h, respectively. Total RNA was extracted and used for qRT-PCR analyses. Results were normalized against ACTIN 2 mRNA levels and expressed as fold change. Asterisks indicate a significant difference (Mann–Whitney U test, **P < 0.01, ***P < 0.01) (from left to right, P = 0.0071, P = 0.03454, P = 0.02066, P = 0.05546, (D) Western blot analysis in 6-day-old seedlings. The specificity of anti-AN antibody was validated using proteins extracted from Col-0, *35S*::*GFP-AN* transgenic plants, and the *an-t1* mutant. (E and F) AN protein levels were decreased after H_2_O_2_ treatment. 6-day-old Col-0 seedlings were treated with or without 100mM H_2_O_2_ for 3h, then the proteins of the mock control (without H_2_O_2_ treatment) and treated Col-0 were extracted, respectively. The anti-AN antibody and anti-Actin antibody were used in the western blot assay (E). Quantification of relative signal intensity (F) showing a significant difference (Mann–Whitney U test, **P < 0.01) (P = 0.00934).
(TIF)

S3 Fig Analysis of O_2_^• –^ and H_2_O_2_ distribution throughout petal development stages 8–14 in WT. (A) Representative confocal images. The left panel shows petal adaxial epidermal cells viewed from the side using propidium iodide (PI)-stained folded petals (stages 8–14). The middle panel shows dihydroethidium (DHE)-stained non-folded petals (stages 8–14) for analysis of O_2_^• –^. The right panel shows CM-H2DCFDA-stained non-folded petals (stages 8–14) for analysis of H_2_O_2_. Scale bars, 20 μm. (B and C) Comparative analysis of O_2_^• –^ (B) and H_2_O_2_ (C) intensity units throughout petal development stages 8–14. For comparative O_2_^• –^ (B) and H_2_O_2_ (C) analysis, a region of interest (ROI) at the adaxial epidermis from WT petals was quantified by ImageJ. Quantitative data are averages ± SD of 30 petals.
(TIF)

S4 Fig Analyses of ROS levels of adaxial epidermal cells in the basal regions of the petal blades. (A) A wild-type mature *A*. *thaliana* petal for observation of adaxial epidermal cell shape. The square area of the basal region of the petal blade visualized by SEM shows relative flat epidermal cell shape. This region was used for the detection of ROS levels over the course of cell development. (B) Confocal images of dihydroethidium (DHE)- and CM-H2DCFDA-stained WT and *an-t1* adaxial epidermal cells from the regions indicated in A. Scale bars = 25 μm. (C and D) Comparative analysis of O_2_^• –^ (C) and H_2_O_2_ (D) intensity units throughout stages 8–14. A region of interest (ROI) at the adaxial epidermal cells from WT and *an-t1* was quantified, respectively, by ImageJ.
(TIF)

S5 Fig Analyses of H_2_O_2_ accumulation in WT and *an* mutants. (A) CM-H2DCFDA-stained non-folded petals (stages 10, 12, and 14) for analysis of H_2_O_2_ in WT, *an-t1*, *an-t2*, and *an-t1 COM#1*. The pseudocolor scale indicates signal intensity. Scale bar, 50 μm. (B) Quantitative analysis of H_2_O_2_ intensity units from WT, *an-t1*, *an-t2*, and *an-t1 COM #1* at indicated petal development stages. The images under the pseudocolor scale were used for the fluorescence intensity measurement and indicate the region of the cell where the fluorescence intensity was measured by ImageJ. Asterisks indicate a significant difference (Mann–Whitney U test, *P < 0.05,**P < 0.01, ***P < 0.001) (from left to right, P = 0.03564, P = 0.0139, P = 0.41413, no significant difference, P = 0.00064, P = 0.00047, P = 0.60387, no significant difference, P = 0.00903, P = 0.00143, P = 0.47984). Values are averages ± SD of 20 petals.
(TIF)

S6 Fig Loss of AN function results in increased ROS levels in cotyledon pavement cells and leaf trichomes. (A) Confocal images of dihydroethidium (DHE)- and CM-H2DCFDA-stained cotyledon pavement cells in WT and *an-t1*. Scale bar = 50 μm. (B) Quantification of fluorescent signal intensity of cotyledon pavement cells. a region of interest (ROI) at the pavement cells was quantified by ImageJ. Mann–Whitney U test, **P < 0.01, ***P < 0.001 (from left to right, P = 0.00222, P = 0.00041). Values are given from 80 cells of 10 cotyledons. (C) Confocal images of dihydroethidium (DHE)- and CM-H2DCFDA-stained leave trichomes in WT and *an-t1*. Scale bar = 100 μm. (D) Quantification of fluorescent signal intensity of trichomes. a region of interest (ROI) at the trichomes was quantified by ImageJ. Mann–Whitney U test, **P < 0.01, ***P < 0.001 (from left to right, P = 0.00001, P = 0.00662). Values are given from 40 trichomes.
(TIF)

S7 Fig Phenotypic analyses of conical cells after time-series treatments for reducing endogenous O_2_^• –^ or H_2_O_2_ levels. (A, D, and G) Representative images of conical cell phenotypes from WT mature flowers. For reduced endogenous O_2_^• –^ or H_2_O_2_, flower buds at development stage 7, stage 9, and stage 11 were treated by mock, 1 mM KI, and 5 mM n-propyl gallate (PG) for one-time treatment, respectively. Mature petals at stage 14 were used for cellular phenotype analyses. Scale bars = 20 μm. (B, C, E, F, H, and I) Quantification of cone height (B, E, and H) and cone angle (C, F, and I) of conical cells. Mann–Whitney U test, ns, no significant difference, **P < 0.01, ***P < 0.001. (B, P = 0.0623, P = 0.00047; C, P = 0.0623, P = 0.00047; E, P = 0.0008, P = 0.00002; F, P = 0.00253, P = 0.00017; H, P = 0.00001, P = 0.00002; I, P = 0.00008, P = 0.00048). For all data sets for quantifications, n = 150 cells form 20 petals.
(TIF)

S8 Fig Analysis of endogenous ROS levels after KI or PG treatment. (A) Representative images of inflorescences after treating with mock solution, KI, and PG. Scale bar = 1 mm. (B and C) DHE (B) or CM-H2DCFDA (C) staining petals for analysis of O_2_^• –^ and H_2_O_2_. WT flower buds at development stage 7 were treated by mock, 5 mM PG, and 1 mM KI, respectively, and the same treatment was repeated four times 24 h later. The flower buds developed into stage 14 mature petals were used for analysis of O_2_^• –^ and H_2_O_2_. Scale bars, 100 μm.
(TIF)

S9 Fig Analysis of H_2_O_2_ accumulation in WT and 35S::*CA*-*ROP2* overexpression lines. (A and B) CM-H2DCFDA-stained petals (stages 10, 12, and 14) for analysis of H_2_O_2_ in WT (A) and 35S::*CA*-*ROP2* line (B). The pseudocolor scale was used to indicate the signal intensity. Scale bar, 50 μm.
(TIF)

S10 Fig Analysis of ROP2 activity in WT, *an-t1*, *an-t2*, and *spk1-4*. (A) Analysis of ROP2 activity. WT and mutant inflorescences were collected and used for protein extraction. The *spk1-4* mutant that was shown to have reduced ROP2 activity is used as a control. The experiment was repeated three times with similar results. (B) Quantification of active ROP2 level. Asterisks indicate a significant difference, Mann–Whitney U test, ***P < 0.001 (P = 0.00044), ns indicating no significant difference (from left to right, P = 0.6525, P = 0.0924).
(TIF)

S11 Fig Analysis of GFP-AN immunoprecipitates. Western blotting analysis of the pull-down samples from GFP lines and GFP-AN lines. Anti-GFP antibody was used for the western blotting analysis, showing high specificity and efficiency for GFP-AN protein enrichment.
(TIF)

S12 Fig Loss of CAT2 function causes a wide-angled tips phenotype in conical cells. (A) Schematic representation of the *CAT2* gene, showing the position of the *cat2-1* mutant allele. The triangle indicates T-DNA insertion. (B) RT-PCR monitoring of *CAT2* mRNA levels in WT and *cat2-1*. *Actin8* mRNA was used as a control. (C and D) Confocal (C) and scanning electron microscope (D) images of conical cells of WT and *cat2-1* from stage 14 flowers. Scale bars = 10 μm. (E and F) Quantification of cone height (E) and cone angle (F) of conical cells. For the boxplots, the box extending from the lower to upper quartile values of the data, with a line representing the data medians. The whiskers extending past 1.5 of the interquartile range. Mann–Whitney U test, ***P < 0.001 (E, P = 0.00072, F, P = 0.00094). For all data sets used for quantifications, n = 120 cells from 10 petals.
(TIF)

S13 Fig Co-immunoprecipitation experiments for investigating AN and CAT2/CAT3 interactions. (A and B) Co-immunoprecipitation experiments for investigating interactions between AN and CAT2 (A), and AN and CAT3 (B). Total proteins were extracted from inflorescences of transgenic lines expressing *35S*::*CAT2-GFP*, *35S*::*CAT3-GFP*, and *35S*::*GFP* (as a control), respectively, and were immunoprecipitated by GFP-Trap agarose beads. The immunoprecipitated complexes were detected by anti-AN antibody. Note that no interactions were found between AN and CAT2/CAT3.
(TIF)

S14 Fig Phenotypic analyses of *an-t1* enhancers. (A) Representative images of 7-day-old seedlings of WT, *ktn1-4*, *ktn1-7*, and *ktn1-8*. Scale bar = 0.5 cm. (B) Quantification of root length from WT, *ktn1-4*, *ktn1-7* and *ktn1-8*. Mann–Whitney U test, **P < 0.01, ***P < 0.001 (from left to right, P = 0.00014, P = 0.00069, P = 0.00623). Values are given as the mean ± SD of 20 seedlings. (C) 25-day- and 40-day-old plants from WT, *ktn1-4*, *ktn1-7*, and *ktn1-8*. Scale bars = 5 cm. (D and E) Comparison of conical cell phenotypes between WT, *an-t1*, *an-t1 ktn1-8*, and *an-t1 ktn1-8* in stage 14 mature petals. Representative confocal images (D) and scanning electron microscope images (E), Scale bars = 10 μm. (F and G) Quantification of conical cell phenotypes from WT, *an-t1*, *an-t1 ktn1-8*, and *an-t1 ktn1-8*. For the quantification of cone height (F), ns indicating no significant difference, Mann–Whitney U test, P > 0.05 (from left to right, P = 0.0949, P = 0.09013, P = 0.0868). For the quantification of cone angle (G) **P < 0.01 (from left to right, P = 0.00073, P = 0.00184, P = 0.00188). Values are given as the mean ± SD of 150 cells of 6 petals from three independent plants. (H) Co-immunoprecipitation experiments for investigating AN and KTN1 interactions *in vivo* by transiently coexpressing *35S*::*GFP-AN* with *35S*::*KTN1-My*c in *Nicotiana benthamiana* leaves. Total protein extracts from leaves transiently coexpressing *35S*::*GFP-AN* and *35S*::*KTN1-My*c or *35S*::*GFP* and *35S*::*KTN1-My*c were immunoprecipitated by GFP-Trap agarose beads, and were detected by anti-Myc antibody.
(TIF)

S15 Fig ROS-dependent regulation of microtubule orientation and conical cell shaping. (A) Comparison of microtubule organization between WT and *CA-ROP2* (A). Visualization of microtubules in conical cells from stage 14 petals of WT and *35S*::*CA-ROP2* stably expressing *GFP-TUA6*. Representative confocal images were generated via surface projections of image stacks at 0.5-μm intervals from the top- down view of adaxial epidermis of non-folded petals. Scale bar = 5 μm. (B) Quantification of microtubule alignment. The microtubule alignment measurement was carried out with "OrientationJ", a ImageJ plug-in, to calculate the directional coherency coefficient of the fibers. A coherency coefficient close to 1 represents a strongly coherent orientation of the microtubules. Mann–Whitney U test, *P < 0.05 (P = 0.03967). Values are given as the mean ± SD of 90 cells from 10 petals. (C) Analysis of microtubule organization after eliminating O_2_^• –^ or H_2_O_2_. Representative confocal images were generated via surface projections of image stacks at 0.5-μm intervals from the top view of adaxial epidermis of non-folded petals. Flower buds at development stage 7 of the *GFP-TUA6* marker line were treated by mock, 5 mM PG, and 1 mM KI, respectively, and the same treatment was repeated 24 h later for another four times. Scale bar = 5 μm. (D) Quantification of microtubule alignment in conical cells. Mann–Whitney U test, **P < 0.01, ***P < 0.001 (from left to right, P = 0.00021, P = 0.0047). Values are given as the mean ± SD of more than 50 cells of 6 petals.
(TIF)

S16 Fig H_2_O_2_ treatments lead to abnormal cotyledon pavement cells, correlating with increased microtubule ordering. (A) Confocal images of cotyledon pavement cells from 5-day-old seedlings. WT seeds were sterilized and then grew on Murashige and Skoog medium agar plates supplemented with 0, 1, 2, 5 mM H_2_O_2_, respectively. 5-day-old seedlings were used for phenotype analyses of pavement cells. Scale bar = 25 μm. (B) A cartoon depicting how the lobe lengths were measured. The distance between the segment midpoint and the vertex of the lobe was measured as the lobe length. (C) Quantification of lobe length of cotyledon pavement cell. Mann–Whitney U test, **P < 0.01, ***P < 0.001 (from left to right, P = 0.00128, P = 0.00196, P = 0.00011). (D) Quantification of cell area. Mann–Whitney U test, *P < 0.05, **P < 0.01 (from left to right, P = 0.02016, P = 0.00578, P = 0.00188). Values are given from more than 50 cells of 10 cotyledons. (E) Visualization of cortical microtubules in pavement cells. Seeds of a transgenic microtubule marker line stably expressing *GFP-MBD* were sterilized and then grew on Murashige and Skoog medium agar plates supplemented with 0, 1, 2, 5 mM H_2_O_2_, respectively. 5-day-old seedlings were used for imaging analyses. Representative confocal images were generated via surface projections of image stacks at 0.5-μm intervals. Scale bar = 25 μm. (F) Quantification of microtubule alignment. The microtubule alignment measurement was carried out with "OrientationJ", a ImageJ plug-in, to calculate the directional coherency coefficient of the fibers. A coherency coefficient close to 1 represents a strongly coherent orientation of the microtubules. For the boxplots, the box extending from the lower to upper quartile values of the data, with a line representing the data medians. The whiskers extending past 1.5 of the interquartile range. Mann–Whitney U test, *P < 0.05, **P < 0.01 (from left to right, P = 0.01042, P = 0.00519, P = 0.01861). Values are given from more than 25 cells of 10 cotyledons.
(TIF)

S17 Fig Phenotypic analyses of trichomes and microtubule arrangements after H_2_O_2_ treatments. (A) Representative images of 15-day-old seedlings with or without H_2_O_2_ treatments. WT seeds were sterilized and then grew on Murashige and Skoog medium agar plates supplemented with 0 and 5 mM H_2_O_2_, respectively. 15-day-old seedlings were used for phenotype analyses. Scale bar = 1 mm. (B) Representative images of trichomes from 15-day-old seedlings with or without H_2_O_2_ treatments. WT seeds were sterilized and then grew on Murashige and Skoog medium agar plates supplemented with 0 and 5 mM H_2_O_2_, respectively. 15-day-old seedlings were used for analysis of trichome phenotype. Scale bar = 100 μm. (C) A cartoon depicting how the trichome length was measured. (D) Trichome branch (br) distribution of mock and H_2_O_2_ treatment. Values are given as the mean ± SD. (E) Quantification of the trichome length. Mann–Whitney U test, *P < 0.05 (P = 0.04255). Values are given from more than 30 trichomes. (F) Visualization of cortical microtubules in trichomes from 15-day-old seedlings with or without H_2_O_2_ treatments. Seeds of a transgenic line stably expressing *GFP-MBD* were sterilized and then grew on Murashige and Skoog medium agar plates supplemented with 0 and 5 mM H_2_O_2_, respectively. Trichomes of 15-day-old seedlings were used for imaging analyses. Scale bar = 10 μm. (G) Quantification of microtubule alignment. The microtubule alignment measurement was carried out with "OrientationJ", a ImageJ plug-in, to calculate the directional coherency coefficient of the fibers. A coherency coefficient close to 1 represents a strongly coherent orientation of the microtubules. For the boxplots, the box extending from the lower to upper quartile values of the data, with a line representing the data medians. The whiskers extending past 1.5 of the interquartile range. Mann–Whitney U test, **P < 0.01 (P = 0.00393). Values are given from more than 20 trichomes.
(TIF)

S18 Fig Visualization of microtubules in conical cells of wild type, *an-t1*, *ktn1-4*, and the *an-t1 ktn1-4* mutant. (A) Visualization of microtubules in conical cells from mature petals. Representative confocal images were generated via surface projections of image stacks at 0.5-μm intervals from the top view of adaxial epidermis of non-folded petals. The microtubule reporter line stably expressing GFP-TUA6 was used for the observation of microtubule arrays. Scale bar = 5 μm. (B) Quantification of microtubule alignment. The microtubule alignment measurement was carried out with "OrientationJ", a ImageJ plug-in, to calculate the directional coherency coefficient of the fibers. A coherency coefficient close to 1 represents a strongly coherent orientation of the microtubules. Mann–Whitney U test, **P < 0.01, ***P < 0.001 (from left to right, P = 0.00759, P = 0.0002, P = 0.00088). Values are given from 40 cells of 10 petals.
(TIF)

S19 Fig Analysis of ROS accumulation in WT and *ktn1-4*. (A) DHE-stained conical cells for O_2_^• –^ analysis of WT and *ktn1-4* from stage 14 flowers. Scale bars = 10 μm. (B) Quantification of DHE-detected O_2_^• –^ in conical cells. For comparative O_2_^• –^ analysis, a region of interest (ROI) at the conical cells was quantified by ImageJ. For the boxplots, the box extending from the lower to upper quartile values of the data, with a line representing the data medians. The whiskers extending past 1.5 of the interquartile range. ns indicating no significant difference from WT (Mann–Whitney U test, P > 0.05). Values are given from 140 cells of 20 petals. (C) CM-H2DCFDA stained conical cells for H_2_O_2_ analysis of WT and *ktn1-4* from stage 14 flowers. Scale bars = 10 μm. (D) Quantification of CM-H2DCFDA-detected H_2_O_2_ in conical cells of WT and *ktn1-4*. For comparative O_2_^• –^ analysis, a region of interest (ROI) at the conical cells was quantified by ImageJ. ns indicating no significant difference from WT (Mann–Whitney U test, P > 0.05). Values are given from 140 cells of 20 petals.
(TIF)

S1 Table Analysis of AN-GFP immunoprecipitates via mass spectrometry (MS). (XLSX)

S2 Table Primers used in this study. (DOCX)
