# Supplementary figures and images for: Reactive oxygen species mediate conical cell shaping in Arabidopsis thaliana petals
Source: PLoS Genet. 2018 Oct 8;14(10):e1007705. doi: 10.1371/journal.pgen.1007705 (PMC6203401; doi:10.1371/journal.pgen.1007705)

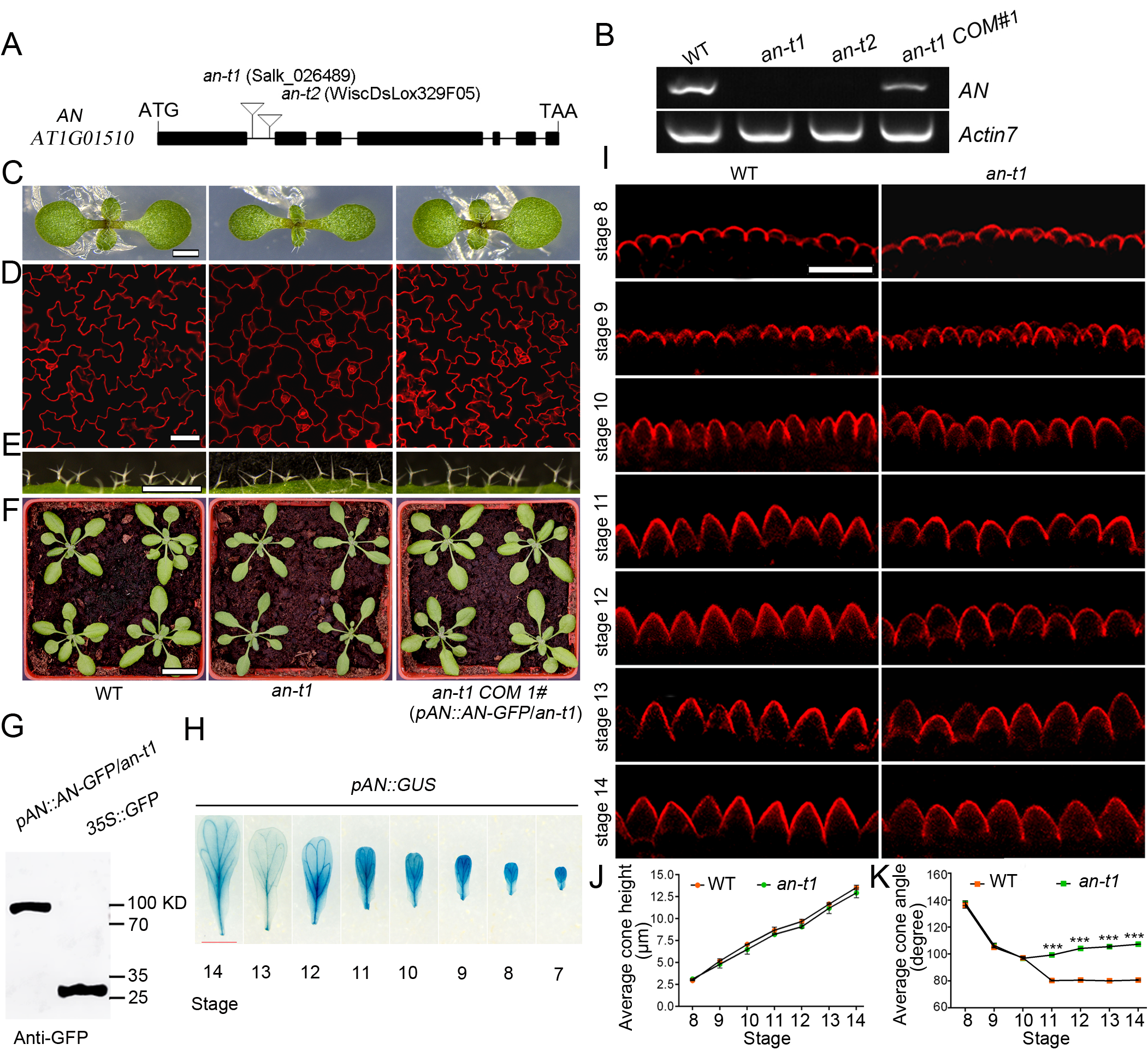

Supplement: S1 Fig — (A) Schematic representation of the AN gene, showing the nature and position of the an-t1 and an-t2 mutant alleles. Triangles indicate T-DNA insertions. (B) RT-PCR analysis of an-t1, an-t2, and an-t1 COM #1. (C–F) Phenotypic analyses of an-t1, an-t2, and an-t1 COM#1. The panels show phenotypes of 5-day-old seedlings (C), cotyledon pavement cells (D), leaf trichomes (E), and 3-week-old plants (F). Scale bars: 1 mm in C and E, 50 μm in D, and 1cm in F. (G) Western blotting analysis of protein expression of the pAN::AN-GFP/an-t1 and 35S::GFP. (H) Analysis of AN promoter activity throughout petal development as monitored in the promoter AN::GUS line. (I) Comparison of conical cells' geometry of wild type and the an-t1 mutant at developmental stages 8–14. Scale bar = 20 μm. (J and K) Quantitative analyses of cone height and angle of conical cells from development stages 8 to 14 in WT and an-t1. Quantification of cone height (J) showed that there were no significant differences between WT and an-t1 at each indicated developmental stages (Mann–Whitney U test, P = 0.41635, P = 0.26494, P = 0.63984, P = 0.12968, P = 0.11702, P = 0.17235, P = 0.14463). Quantification of cone height (K) showed that, at development stages 8 to 10, an-t1 conical cells’ cone angles were similar to WT at stages 8–10 (Mann–Whitney U test, P = 0.22442, P = 0.19294, P = 0.18819) whereas at development stage 11 and beyond, an-t1 displayed significantly increased cone angles compared with WT (***P < 0.001, Mann–Whitney U test) (from left to right, P = 0.00009, P = 0.00006, P = 0.00033, P = 0.00006). Values are given as mean ± SD of more than 180 cells of 10 petals from independent plants. (TIF) [file pgen.1007705.s001.tif]

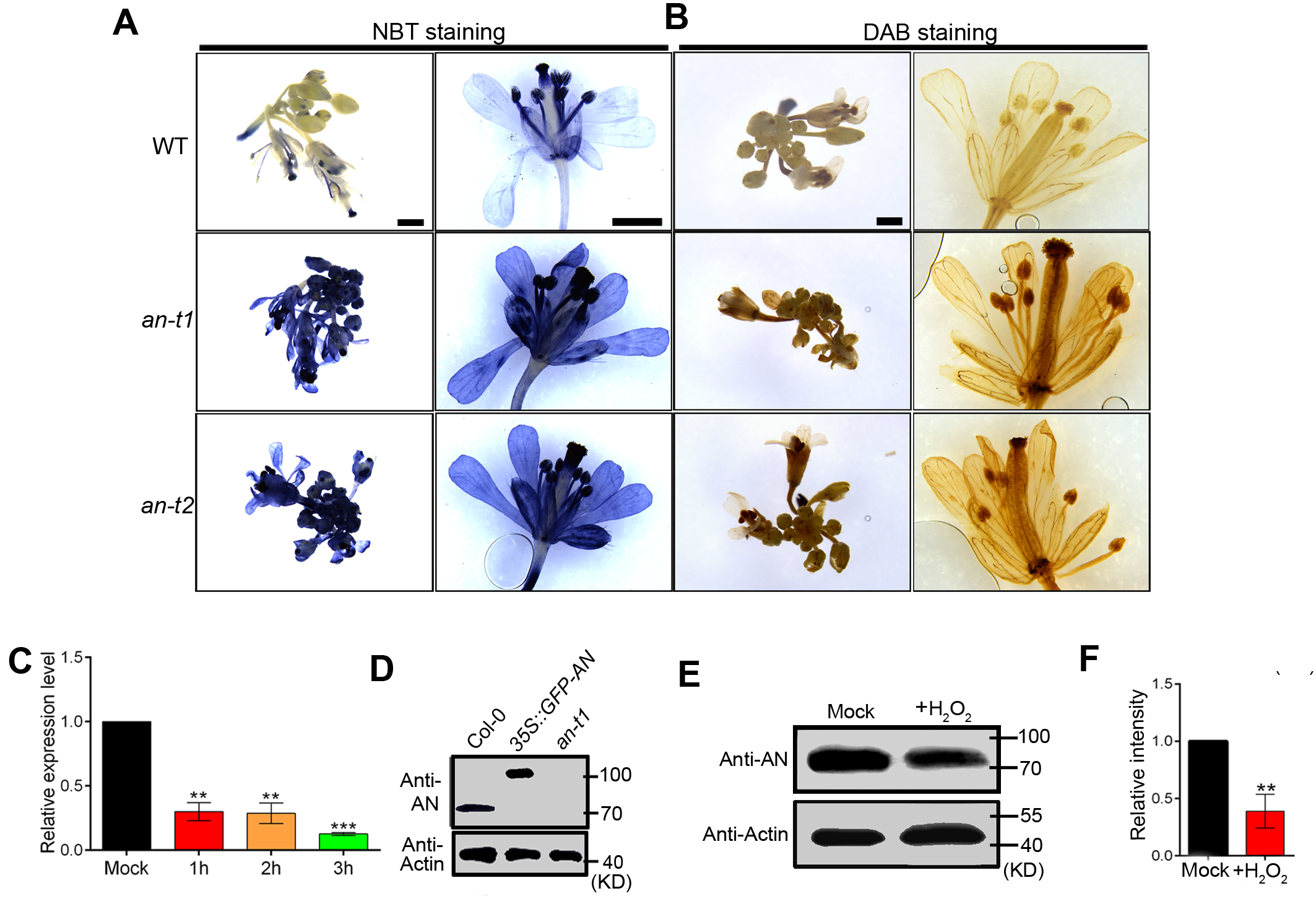

Supplement: S2 Fig — (A) NBT staining for superoxide in WT and an-t1 inflorescences and stage 14 flowers. an-t1 had higher levels of superoxide than WT. Scale bars = 1 mm. (B) DAB staining for H2O2 in WT and an-t1 inflorescences and stage 14 flowers. an-t1 had higher levels of H2O2 than WT. Scale bars = 1 mm. (C) AN mRNA levels were decreased after H2O2 treatment. 6-day seedlings of Col-0 were treated with 100 mM H2O2 for 0h (mock), 1h, 2h, and 3h, respectively. Total RNA was extracted and used for qRT-PCR analyses. Results were normalized against ACTIN 2 mRNA levels and expressed as fold change. Asterisks indicate a significant difference (Mann–Whitney U test, **P < 0.01, ***P < 0.01) (from left to right, P = 0.0071, P = 0.03454, P = 0.02066, P = 0.05546, (D) Western blot analysis in 6-day-old seedlings. The specificity of anti-AN antibody was validated using proteins extracted from Col-0, 35S::GFP-AN transgenic plants, and the an-t1 mutant. (E and F) AN protein levels were decreased after H2O2 treatment. 6-day-old Col-0 seedlings were treated with or without 100mM H2O2 for 3h, then the proteins of the mock control (without H2O2 treatment) and treated Col-0 were extracted, respectively. The anti-AN antibody and anti-Actin antibody were used in the western blot assay (E). Quantification of relative signal intensity (F) showing a significant difference (Mann–Whitney U test, **P < 0.01) (P = 0.00934). (TIF) [file pgen.1007705.s002.tif]

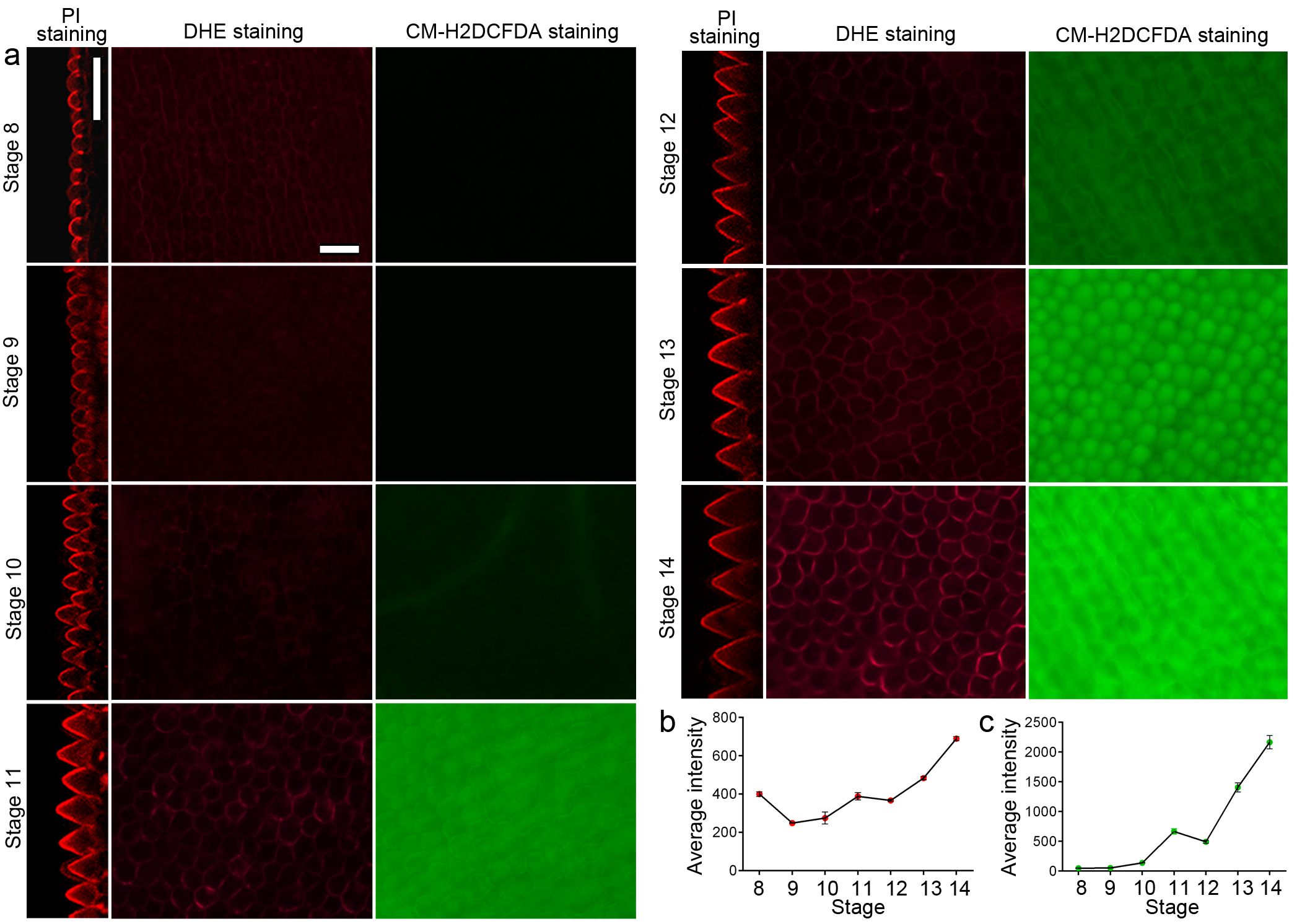

Supplement: S3 Fig — (A) Representative confocal images. The left panel shows petal adaxial epidermal cells viewed from the side using propidium iodide (PI)-stained folded petals (stages 8–14). The middle panel shows dihydroethidium (DHE)-stained non-folded petals (stages 8–14) for analysis of O2• –. The right panel shows CM-H2DCFDA-stained non-folded petals (stages 8–14) for analysis of H2O2. Scale bars, 20 μm. (B and C) Comparative analysis of O2• – (B) and H2O2 (C) intensity units throughout petal development stages 8–14. For comparative O2• – (B) and H2O2 (C) analysis, a region of interest (ROI) at the adaxial epidermis from WT petals was quantified by ImageJ. Quantitative data are averages ± SD of 30 petals. (TIF) [file pgen.1007705.s003.tif]

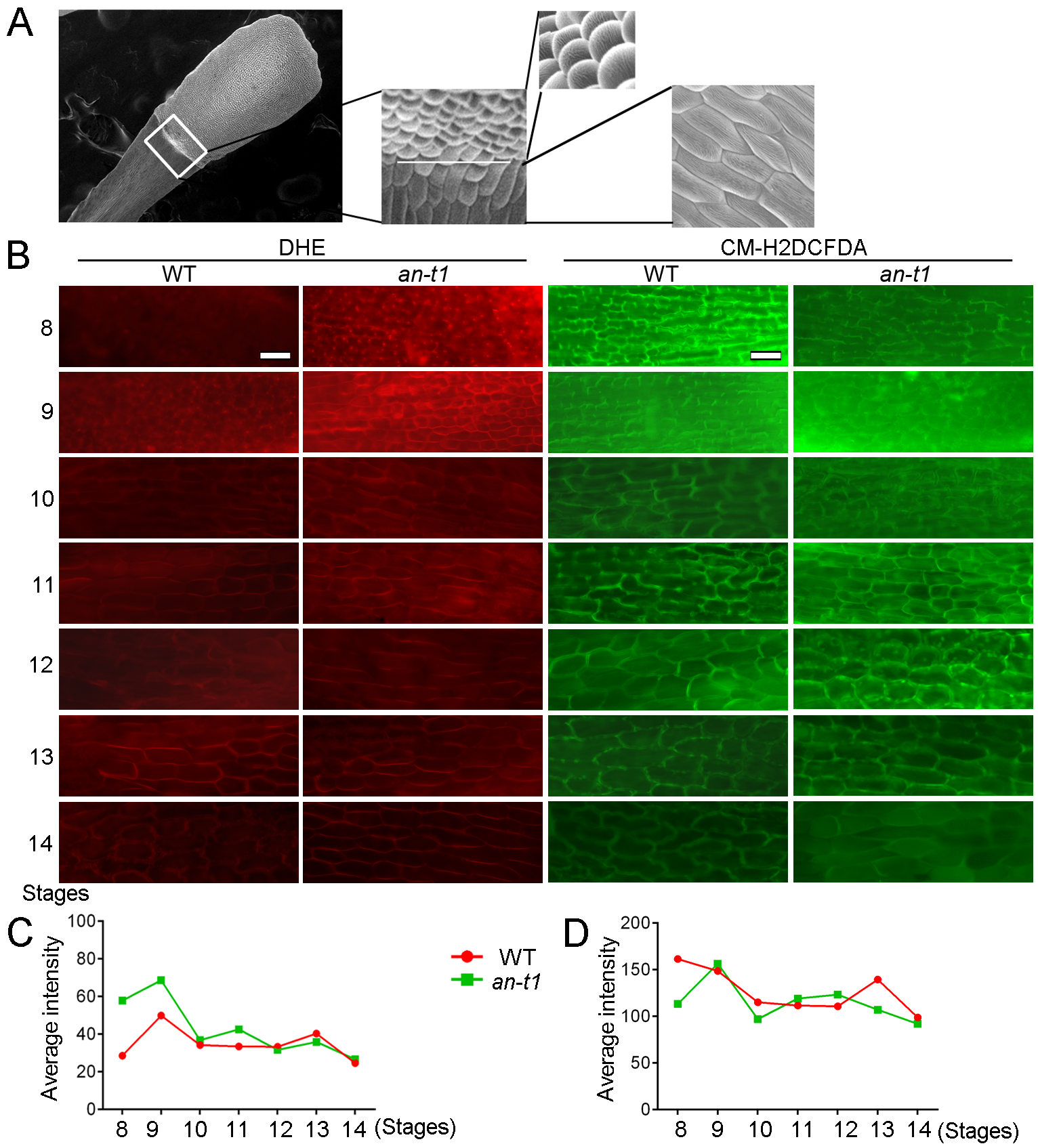

Supplement: S4 Fig — (A) A wild-type mature A. thaliana petal for observation of adaxial epidermal cell shape. The square area of the basal region of the petal blade visualized by SEM shows relative flat epidermal cell shape. This region was used for the detection of ROS levels over the course of cell development. (B) Confocal images of dihydroethidium (DHE)- and CM-H2DCFDA-stained WT and an-t1 adaxial epidermal cells from the regions indicated in A. Scale bars = 25 μm. (C and D) Comparative analysis of O2• – (C) and H2O2 (D) intensity units throughout stages 8–14. A region of interest (ROI) at the adaxial epidermal cells from WT and an-t1 was quantified, respectively, by ImageJ. (TIF) [file pgen.1007705.s004.tif]

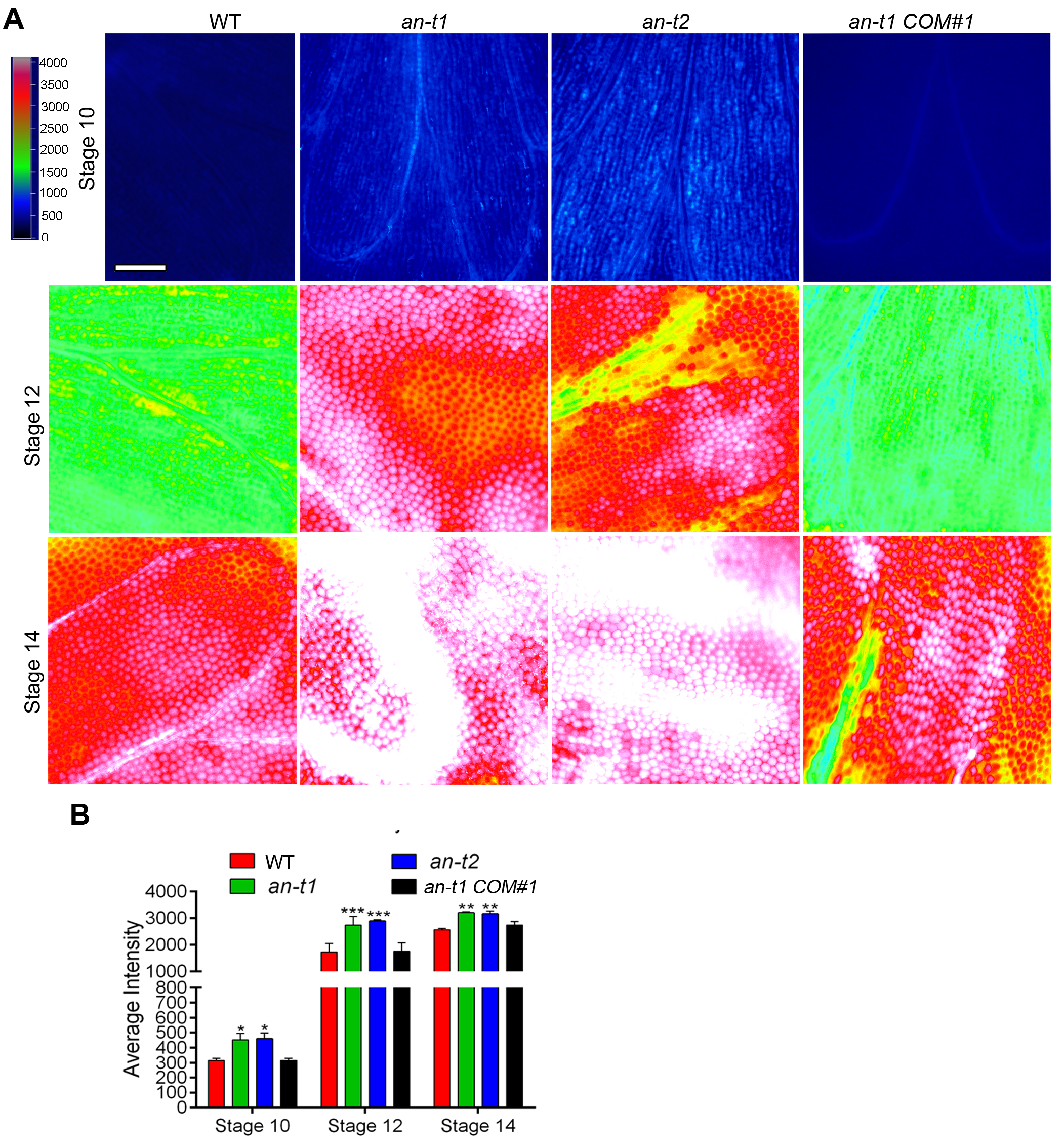

Supplement: S5 Fig — (A) CM-H2DCFDA-stained non-folded petals (stages 10, 12, and 14) for analysis of H2O2 in WT, an-t1, an-t2, and an-t1 COM#1. The pseudocolor scale indicates signal intensity. Scale bar, 50 μm. (B) Quantitative analysis of H2O2 intensity units from WT, an-t1, an-t2, and an-t1 COM #1 at indicated petal development stages. The images under the pseudocolor scale were used for the fluorescence intensity measurement and indicate the region of the cell where the fluorescence intensity was measured by ImageJ. Asterisks indicate a significant difference (Mann–Whitney U test, *P < 0.05,**P < 0.01, ***P < 0.001) (from left to right, P = 0.03564, P = 0.0139, P = 0.41413, no significant difference, P = 0.00064, P = 0.00047, P = 0.60387, no significant difference, P = 0.00903, P = 0.00143, P = 0.47984). Values are averages ± SD of 20 petals. (TIF) [file pgen.1007705.s005.tif]

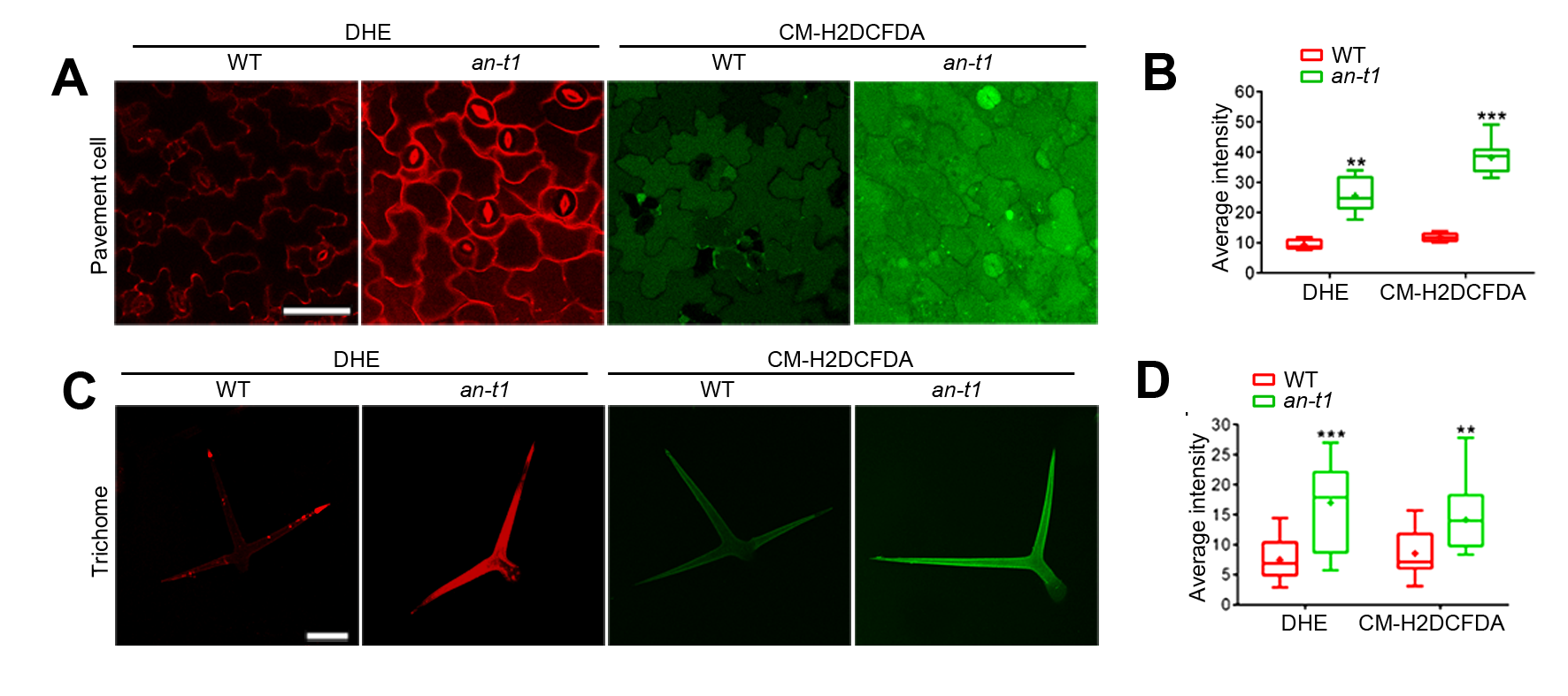

Supplement: S6 Fig — (A) Confocal images of dihydroethidium (DHE)- and CM-H2DCFDA-stained cotyledon pavement cells in WT and an-t1. Scale bar = 50 μm. (B) Quantification of fluorescent signal intensity of cotyledon pavement cells. a region of interest (ROI) at the pavement cells was quantified by ImageJ. Mann–Whitney U test, **P < 0.01, ***P < 0.001 (from left to right, P = 0.00222, P = 0.00041). Values are given from 80 cells of 10 cotyledons. (C) Confocal images of dihydroethidium (DHE)- and CM-H2DCFDA-stained leave trichomes in WT and an-t1. Scale bar = 100 μm. (D) Quantification of fluorescent signal intensity of trichomes. a region of interest (ROI) at the trichomes was quantified by ImageJ. Mann–Whitney U test, **P < 0.01, ***P < 0.001 (from left to right, P = 0.00001, P = 0.00662). Values are given from 40 trichomes. (TIF) [file pgen.1007705.s006.tif]

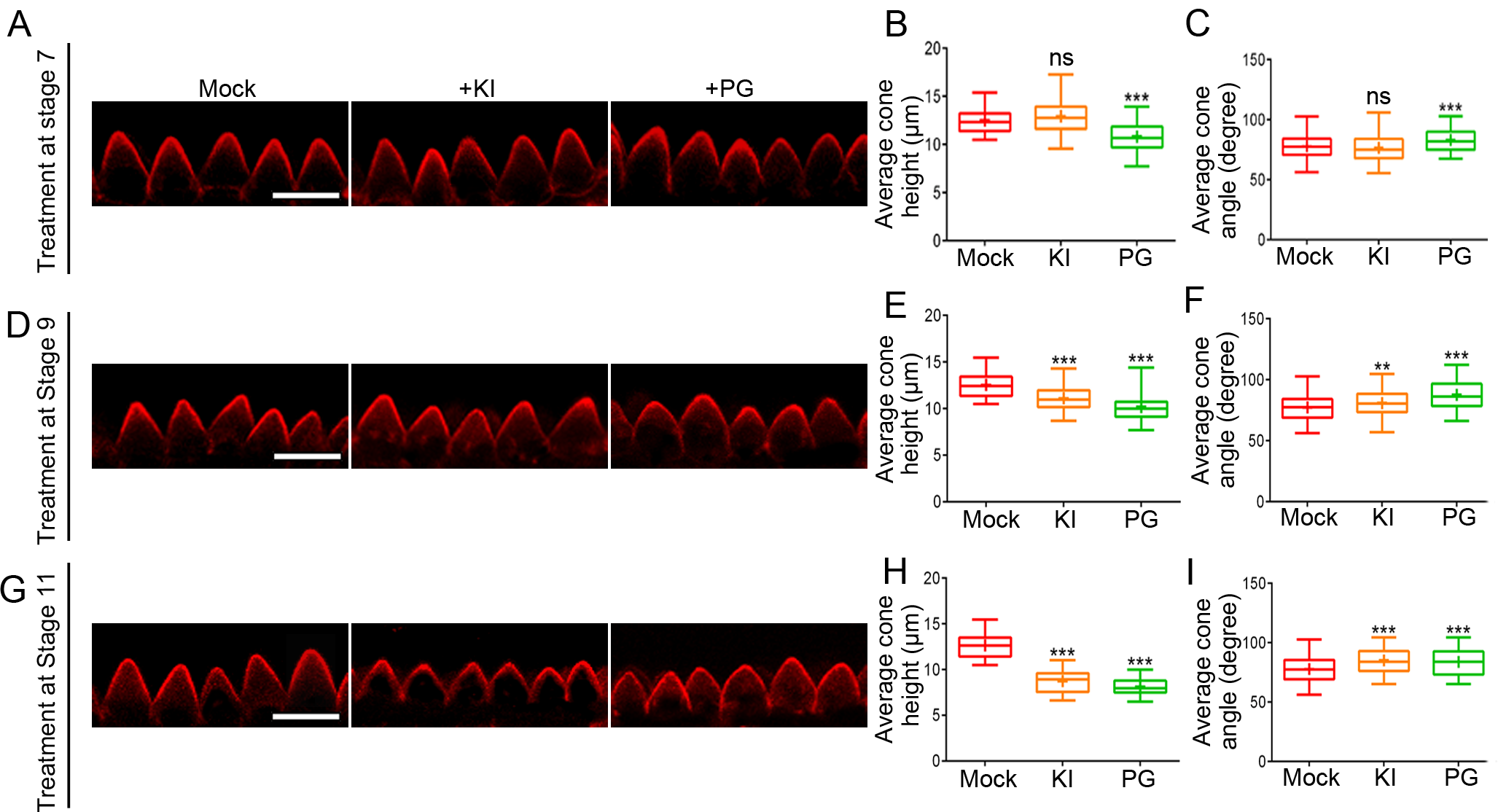

Supplement: S7 Fig — (A, D, and G) Representative images of conical cell phenotypes from WT mature flowers. For reduced endogenous O2• – or H2O2, flower buds at development stage 7, stage 9, and stage 11 were treated by mock, 1 mM KI, and 5 mM n-propyl gallate (PG) for one-time treatment, respectively. Mature petals at stage 14 were used for cellular phenotype analyses. Scale bars = 20 μm. (B, C, E, F, H, and I) Quantification of cone height (B, E, and H) and cone angle (C, F, and I) of conical cells. Mann–Whitney U test, ns, no significant difference, **P < 0.01, ***P < 0.001. (B, P = 0.0623, P = 0.00047; C, P = 0.0623, P = 0.00047; E, P = 0.0008, P = 0.00002; F, P = 0.00253, P = 0.00017; H, P = 0.00001, P = 0.00002; I, P = 0.00008, P = 0.00048). For all data sets for quantifications, n = 150 cells form 20 petals. (TIF) [file pgen.1007705.s007.tif]

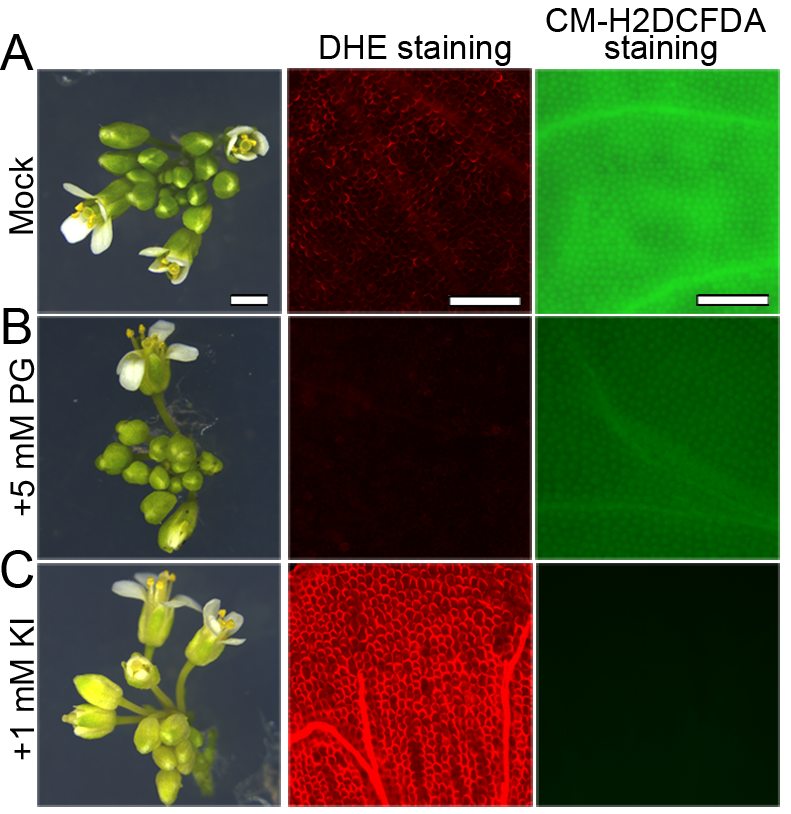

Supplement: S8 Fig — (A) Representative images of inflorescences after treating with mock solution, KI, and PG. Scale bar = 1 mm. (B and C) DHE (B) or CM-H2DCFDA (C) staining petals for analysis of O2• – and H2O2. WT flower buds at development stage 7 were treated by mock, 5 mM PG, and 1 mM KI, respectively, and the same treatment was repeated four times 24 h later. The flower buds developed into stage 14 mature petals were used for analysis of O2• – and H2O2. Scale bars, 100 μm. (TIF) [file pgen.1007705.s008.tif]

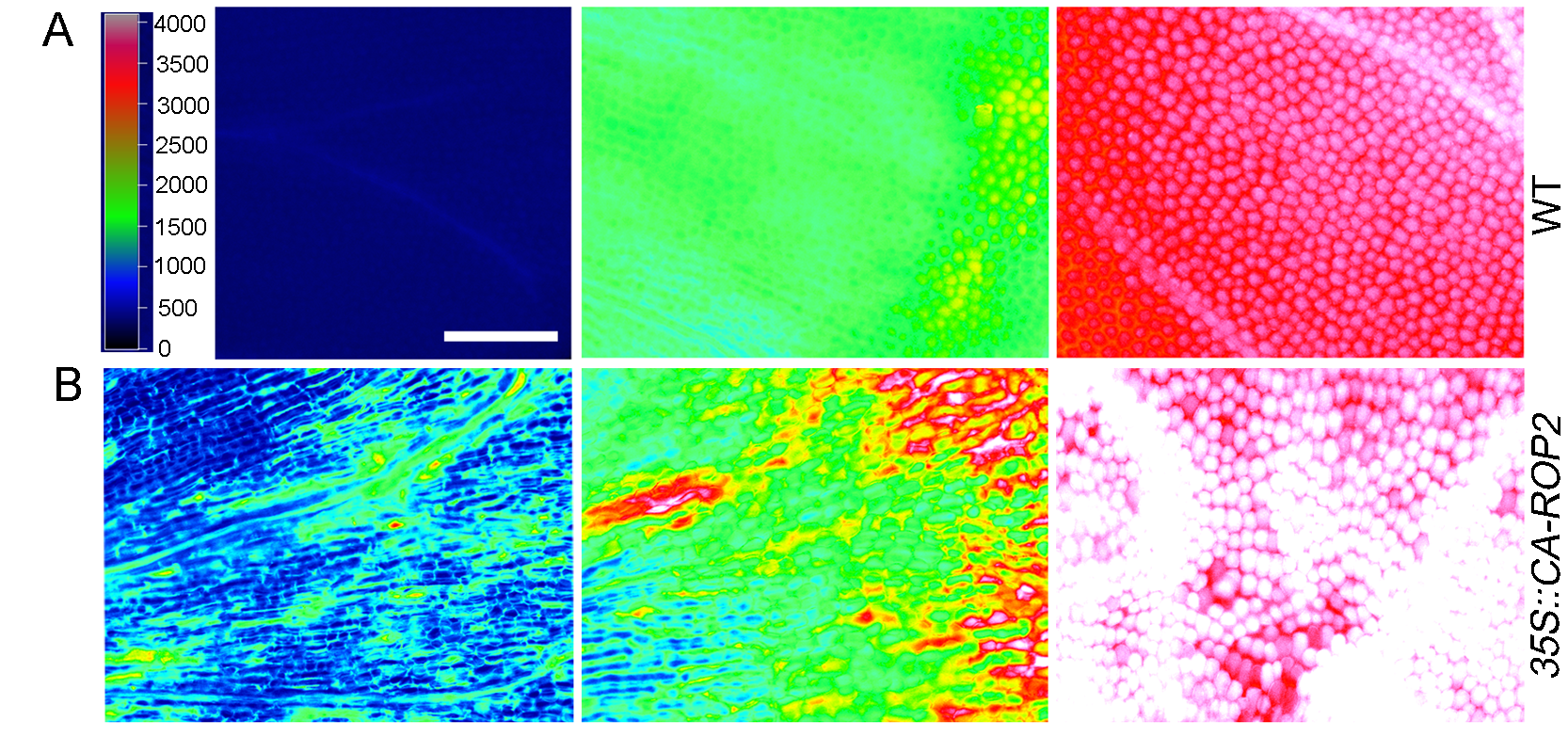

Supplement: S9 Fig — (A and B) CM-H2DCFDA-stained petals (stages 10, 12, and 14) for analysis of H2O2 in WT (A) and 35S::CA-ROP2 line (B). The pseudocolor scale was used to indicate the signal intensity. Scale bar, 50 μm. (TIF) [file pgen.1007705.s009.tif]

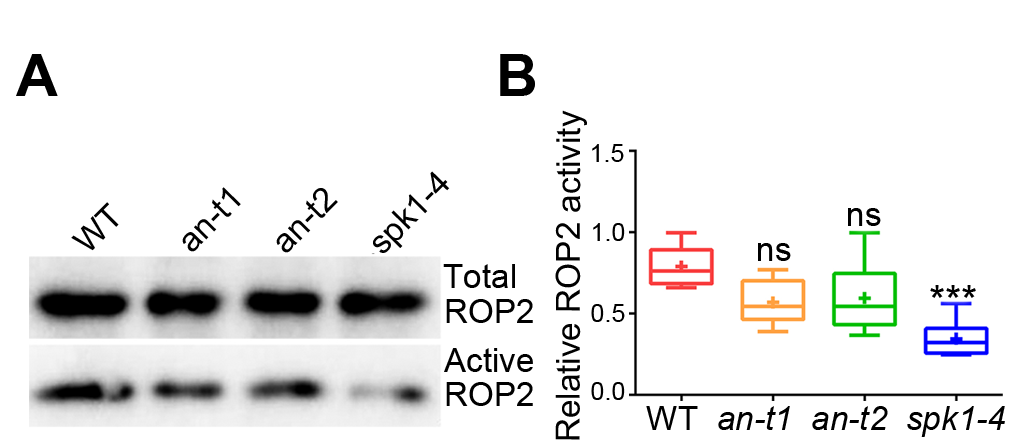

Supplement: S10 Fig — (A) Analysis of ROP2 activity. WT and mutant inflorescences were collected and used for protein extraction. The spk1-4 mutant that was shown to have reduced ROP2 activity is used as a control. The experiment was repeated three times with similar results. (B) Quantification of active ROP2 level. Asterisks indicate a significant difference, Mann–Whitney U test, ***P < 0.001 (P = 0.00044), ns indicating no significant difference (from left to right, P = 0.6525, P = 0.0924). (TIF) [file pgen.1007705.s010.tif]

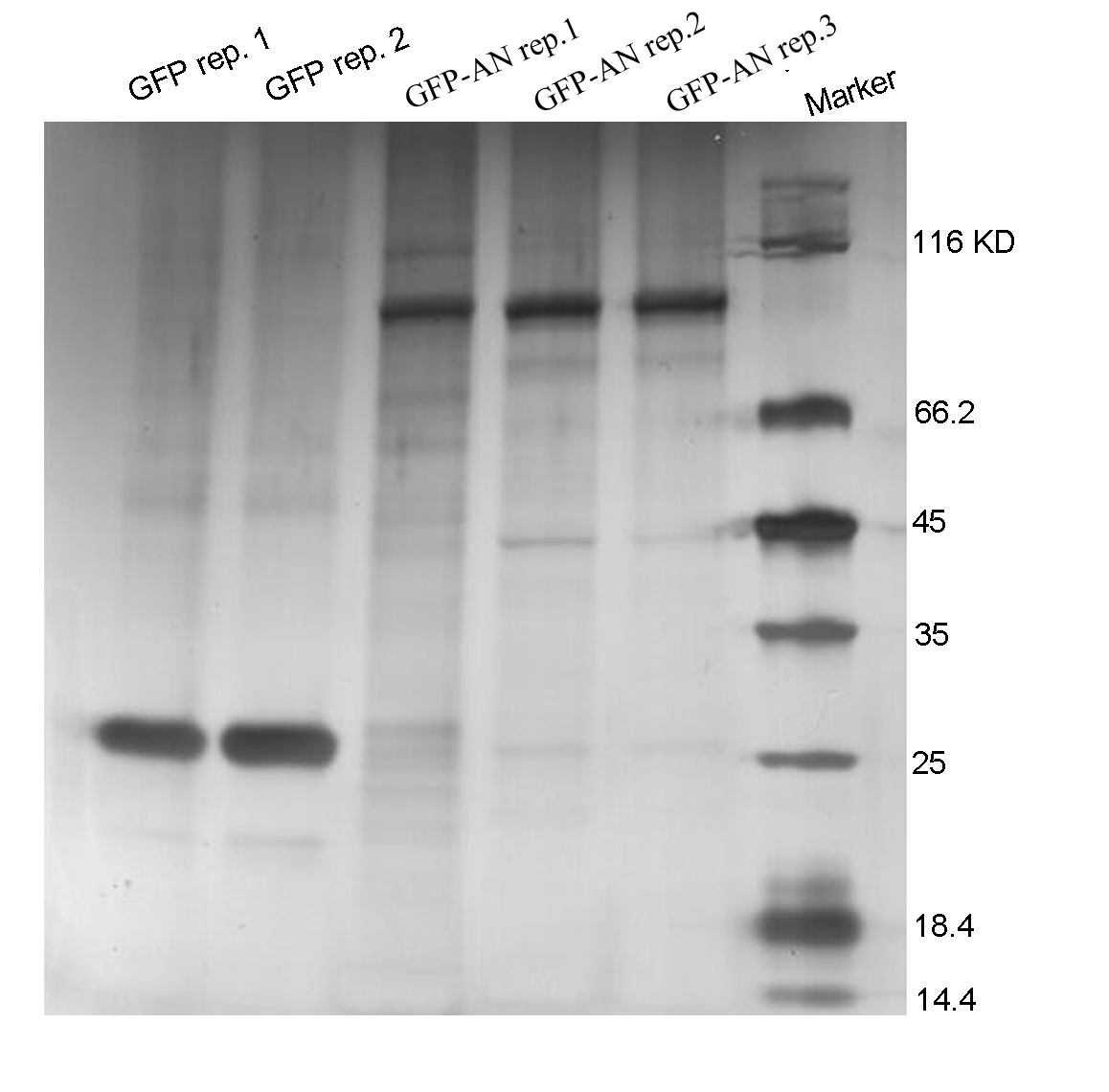

Supplement: S11 Fig — Western blotting analysis of the pull-down samples from GFP lines and GFP-AN lines. Anti-GFP antibody was used for the western blotting analysis, showing high specificity and efficiency for GFP-AN protein enrichment. (TIF) [file pgen.1007705.s011.tif]

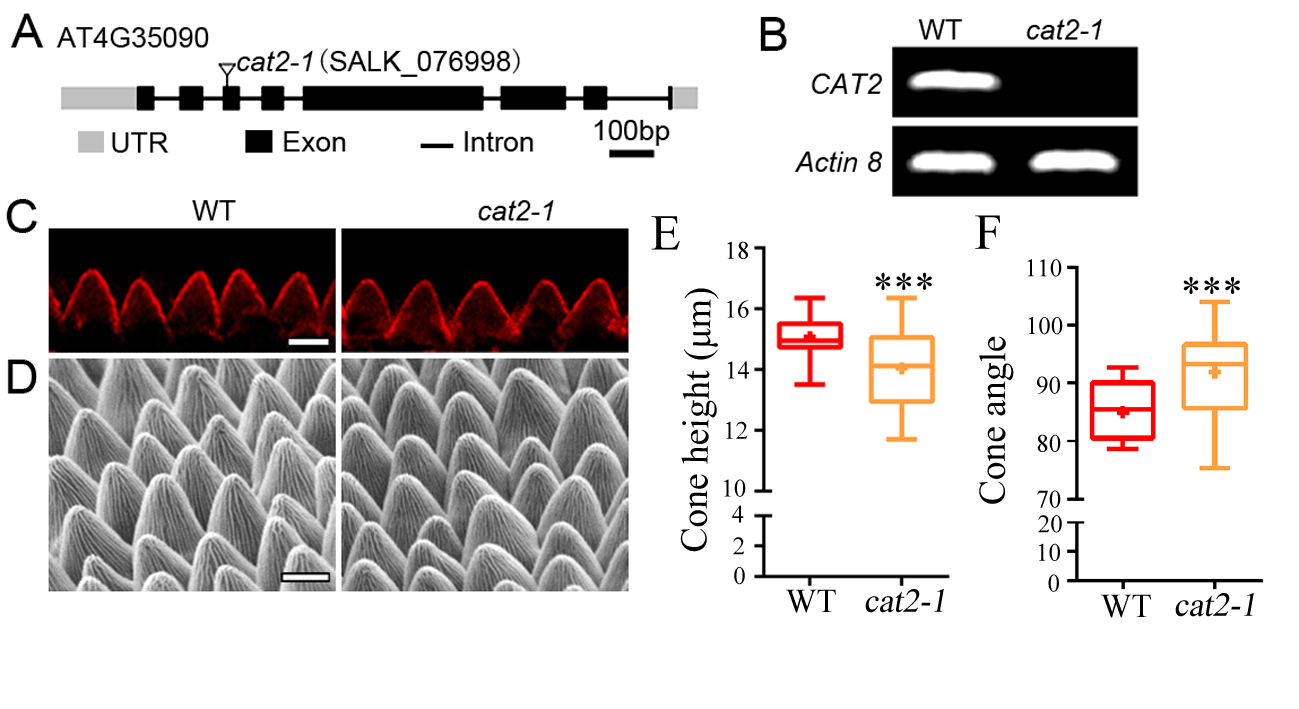

Supplement: S12 Fig — (A) Schematic representation of the CAT2 gene, showing the position of the cat2-1 mutant allele. The triangle indicates T-DNA insertion. (B) RT-PCR monitoring of CAT2 mRNA levels in WT and cat2-1. Actin8 mRNA was used as a control. (C and D) Confocal (C) and scanning electron microscope (D) images of conical cells of WT and cat2-1 from stage 14 flowers. Scale bars = 10 μm. (E and F) Quantification of cone height (E) and cone angle (F) of conical cells. For the boxplots, the box extending from the lower to upper quartile values of the data, with a line representing the data medians. The whiskers extending past 1.5 of the interquartile range. Mann–Whitney U test, ***P < 0.001 (E, P = 0.00072, F, P = 0.00094). For all data sets used for quantifications, n = 120 cells from 10 petals. (TIF) [file pgen.1007705.s012.tif]

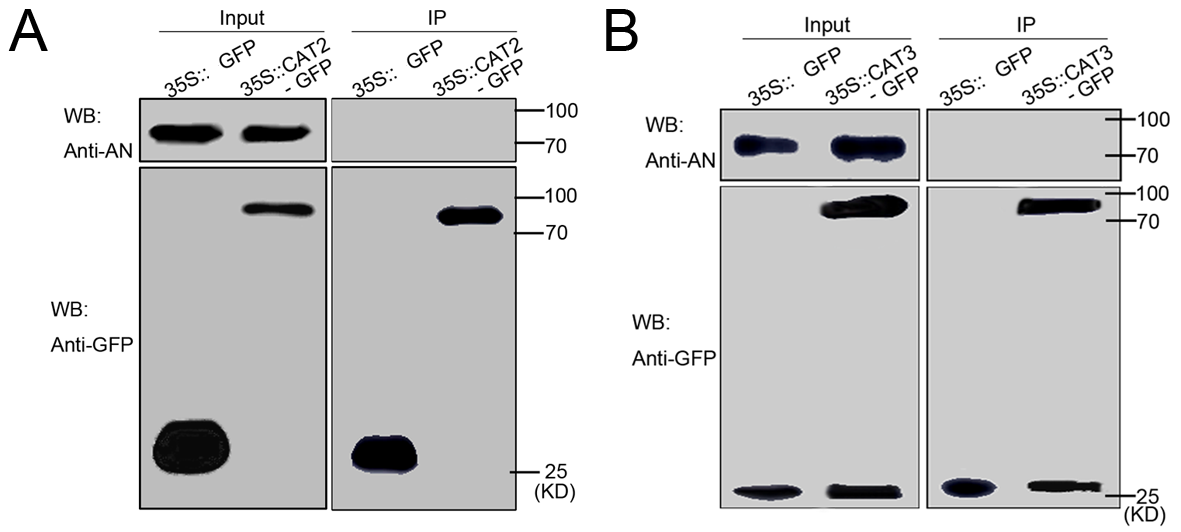

Supplement: S13 Fig — (A and B) Co-immunoprecipitation experiments for investigating interactions between AN and CAT2 (A), and AN and CAT3 (B). Total proteins were extracted from inflorescences of transgenic lines expressing 35S::CAT2-GFP, 35S::CAT3-GFP, and 35S::GFP (as a control), respectively, and were immunoprecipitated by GFP-Trap agarose beads. The immunoprecipitated complexes were detected by anti-AN antibody. Note that no interactions were found between AN and CAT2/CAT3. (TIF) [file pgen.1007705.s013.tif]

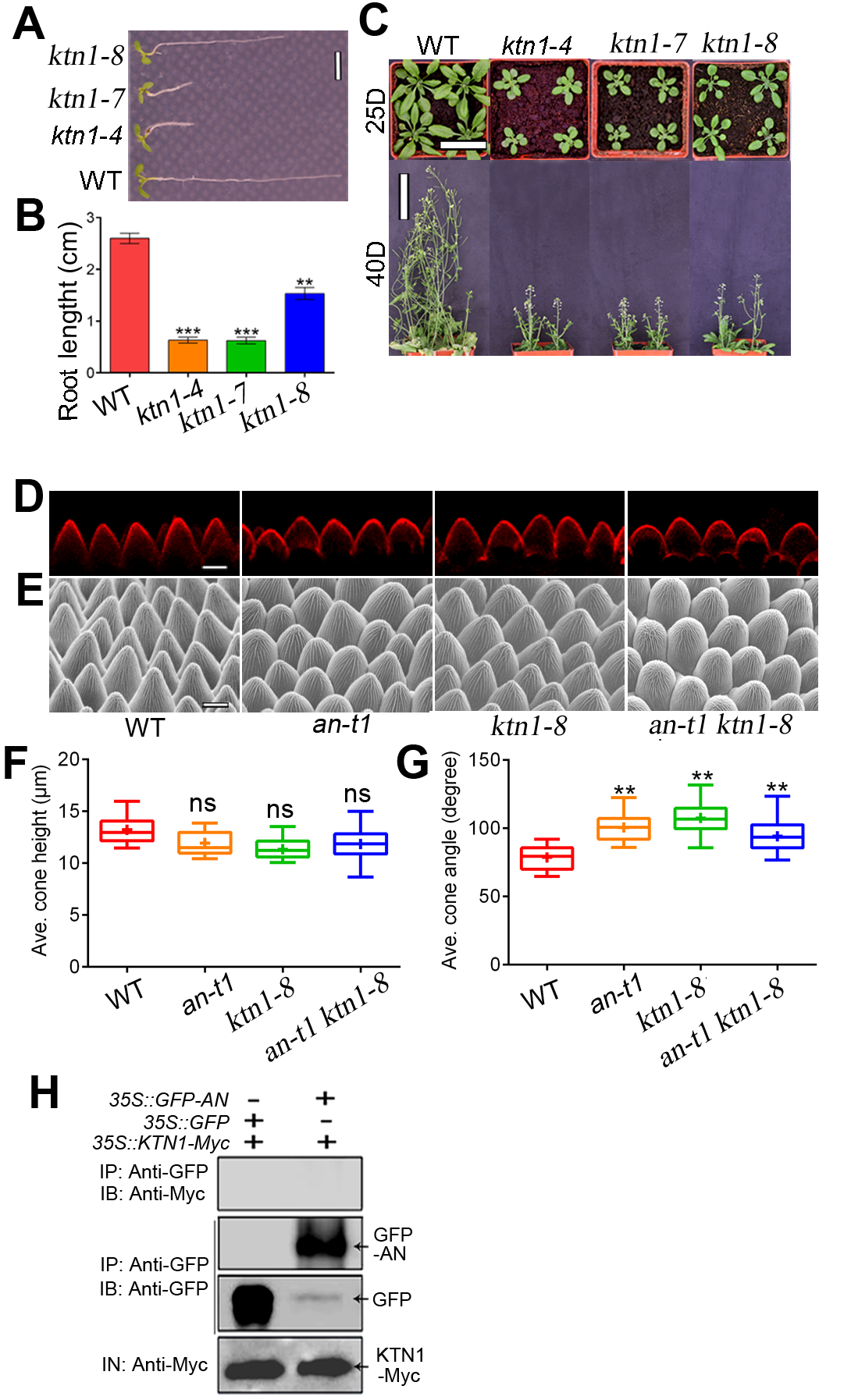

Supplement: S14 Fig — (A) Representative images of 7-day-old seedlings of WT, ktn1-4, ktn1-7, and ktn1-8. Scale bar = 0.5 cm. (B) Quantification of root length from WT, ktn1-4, ktn1-7 and ktn1-8. Mann–Whitney U test, **P < 0.01, ***P < 0.001 (from left to right, P = 0.00014, P = 0.00069, P = 0.00623). Values are given as the mean ± SD of 20 seedlings. (C) 25-day- and 40-day-old plants from WT, ktn1-4, ktn1-7, and ktn1-8. Scale bars = 5 cm. (D and E) Comparison of conical cell phenotypes between WT, an-t1, an-t1 ktn1-8, and an-t1 ktn1-8 in stage 14 mature petals. Representative confocal images (D) and scanning electron microscope images (E), Scale bars = 10 μm. (F and G) Quantification of conical cell phenotypes from WT, an-t1, an-t1 ktn1-8, and an-t1 ktn1-8. For the quantification of cone height (F), ns indicating no significant difference, Mann–Whitney U test, P > 0.05 (from left to right, P = 0.0949, P = 0.09013, P = 0.0868). For the quantification of cone angle (G) **P < 0.01 (from left to right, P = 0.00073, P = 0.00184, P = 0.00188). Values are given as the mean ± SD of 150 cells of 6 petals from three independent plants. (H) Co-immunoprecipitation experiments for investigating AN and KTN1 interactions in vivo by transiently coexpressing 35S::GFP-AN with 35S::KTN1-Myc in Nicotiana benthamiana leaves. Total protein extracts from leaves transiently coexpressing 35S::GFP-AN and 35S::KTN1-Myc or 35S::GFP and 35S::KTN1-Myc were immunoprecipitated by GFP-Trap agarose beads, and were detected by anti-Myc antibody. (TIF) [file pgen.1007705.s014.tif]

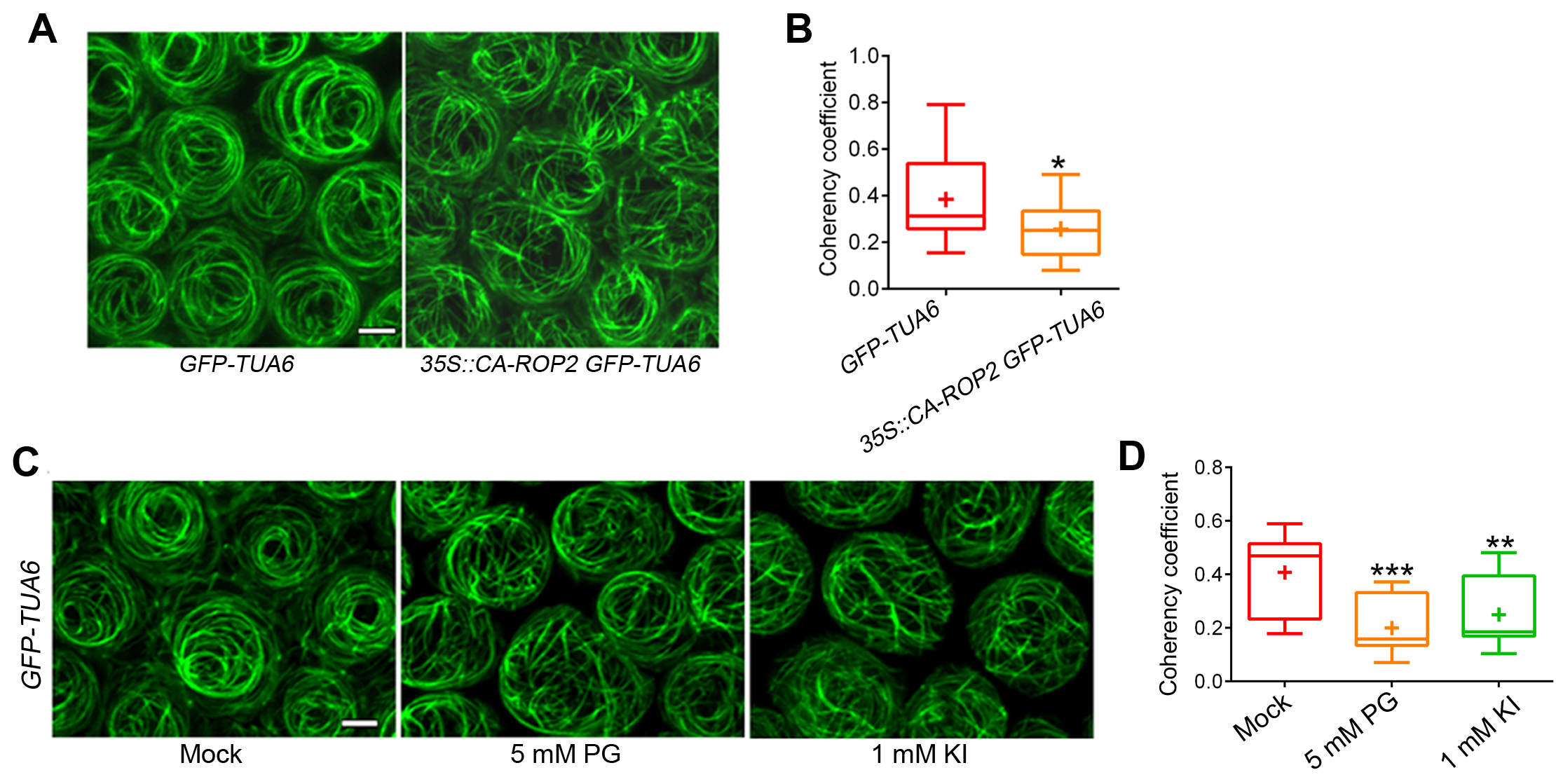

Supplement: S15 Fig — (A) Comparison of microtubule organization between WT and CA-ROP2 (A). Visualization of microtubules in conical cells from stage 14 petals of WT and 35S::CA-ROP2 stably expressing GFP-TUA6. Representative confocal images were generated via surface projections of image stacks at 0.5-μm intervals from the top- down view of adaxial epidermis of non-folded petals. Scale bar = 5 μm. (B) Quantification of microtubule alignment. The microtubule alignment measurement was carried out with "OrientationJ", a ImageJ plug-in, to calculate the directional coherency coefficient of the fibers. A coherency coefficient close to 1 represents a strongly coherent orientation of the microtubules. Mann–Whitney U test, *P < 0.05 (P = 0.03967). Values are given as the mean ± SD of 90 cells from 10 petals. (C) Analysis of microtubule organization after eliminating O2• – or H2O2. Representative confocal images were generated via surface projections of image stacks at 0.5-μm intervals from the top view of adaxial epidermis of non-folded petals. Flower buds at development stage 7 of the GFP-TUA6 marker line were treated by mock, 5 mM PG, and 1 mM KI, respectively, and the same treatment was repeated 24 h later for another four times. Scale bar = 5 μm. (D) Quantification of microtubule alignment in conical cells. Mann–Whitney U test, **P < 0.01, ***P < 0.001 (from left to right, P = 0.00021, P = 0.0047). Values are given as the mean ± SD of more than 50 cells of 6 petals. (TIF) [file pgen.1007705.s015.tif]

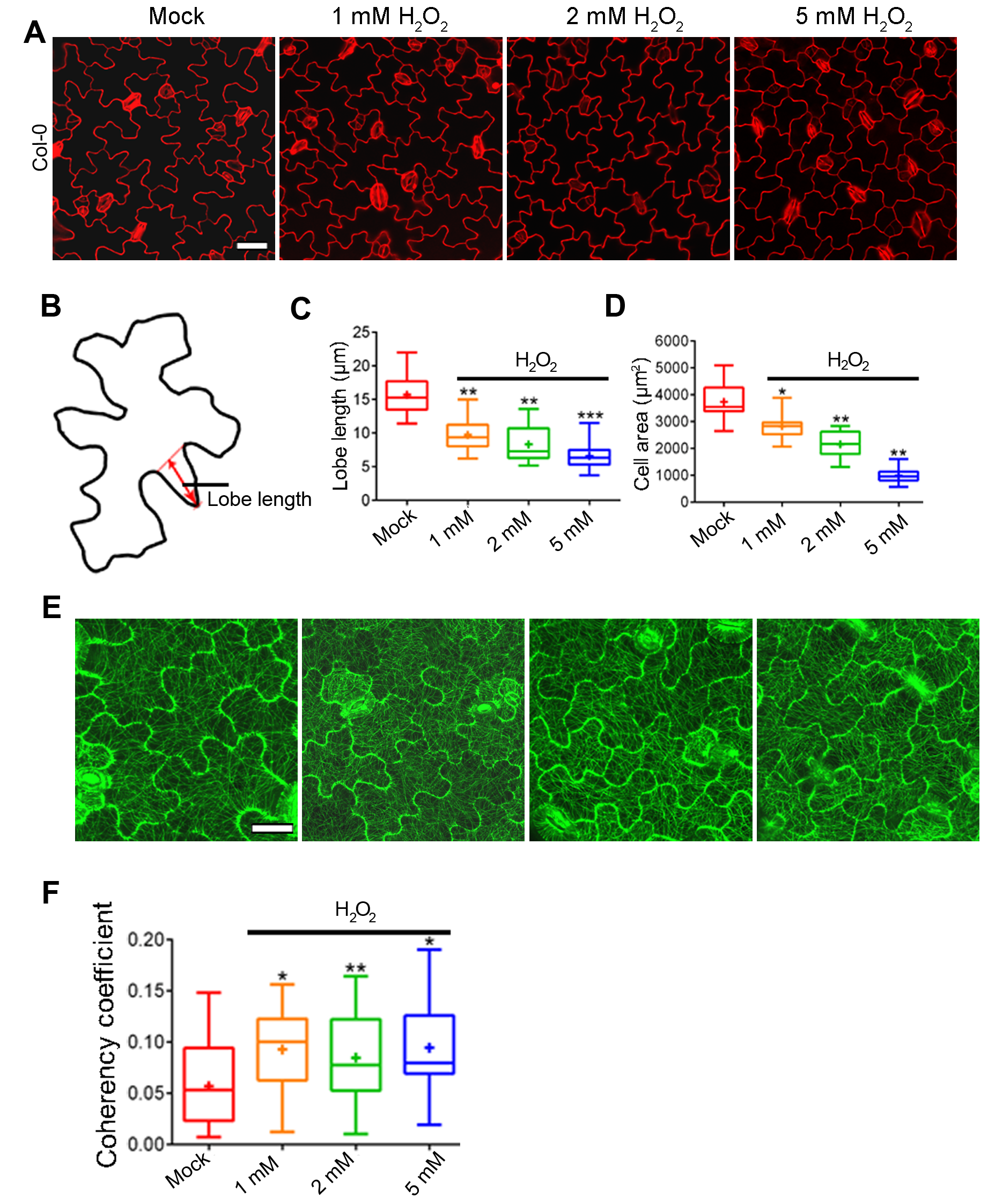

Supplement: S16 Fig — (A) Confocal images of cotyledon pavement cells from 5-day-old seedlings. WT seeds were sterilized and then grew on Murashige and Skoog medium agar plates supplemented with 0, 1, 2, 5 mM H2O2, respectively. 5-day-old seedlings were used for phenotype analyses of pavement cells. Scale bar = 25 μm. (B) A cartoon depicting how the lobe lengths were measured. The distance between the segment midpoint and the vertex of the lobe was measured as the lobe length. (C) Quantification of lobe length of cotyledon pavement cell. Mann–Whitney U test, **P < 0.01, ***P < 0.001 (from left to right, P = 0.00128, P = 0.00196, P = 0.00011). (D) Quantification of cell area. Mann–Whitney U test, *P < 0.05, **P < 0.01 (from left to right, P = 0.02016, P = 0.00578, P = 0.00188). Values are given from more than 50 cells of 10 cotyledons. (E) Visualization of cortical microtubules in pavement cells. Seeds of a transgenic microtubule marker line stably expressing GFP-MBD were sterilized and then grew on Murashige and Skoog medium agar plates supplemented with 0, 1, 2, 5 mM H2O2, respectively. 5-day-old seedlings were used for imaging analyses. Representative confocal images were generated via surface projections of image stacks at 0.5-μm intervals. Scale bar = 25 μm. (F) Quantification of microtubule alignment. The microtubule alignment measurement was carried out with "OrientationJ", a ImageJ plug-in, to calculate the directional coherency coefficient of the fibers. A coherency coefficient close to 1 represents a strongly coherent orientation of the microtubules. For the boxplots, the box extending from the lower to upper quartile values of the data, with a line representing the data medians. The whiskers extending past 1.5 of the interquartile range. Mann–Whitney U test, *P < 0.05, **P < 0.01 (from left to right, P = 0.01042, P = 0.00519, P = 0.01861). Values are given from more than 25 cells of 10 cotyledons. (TIF) [file pgen.1007705.s016.tif]

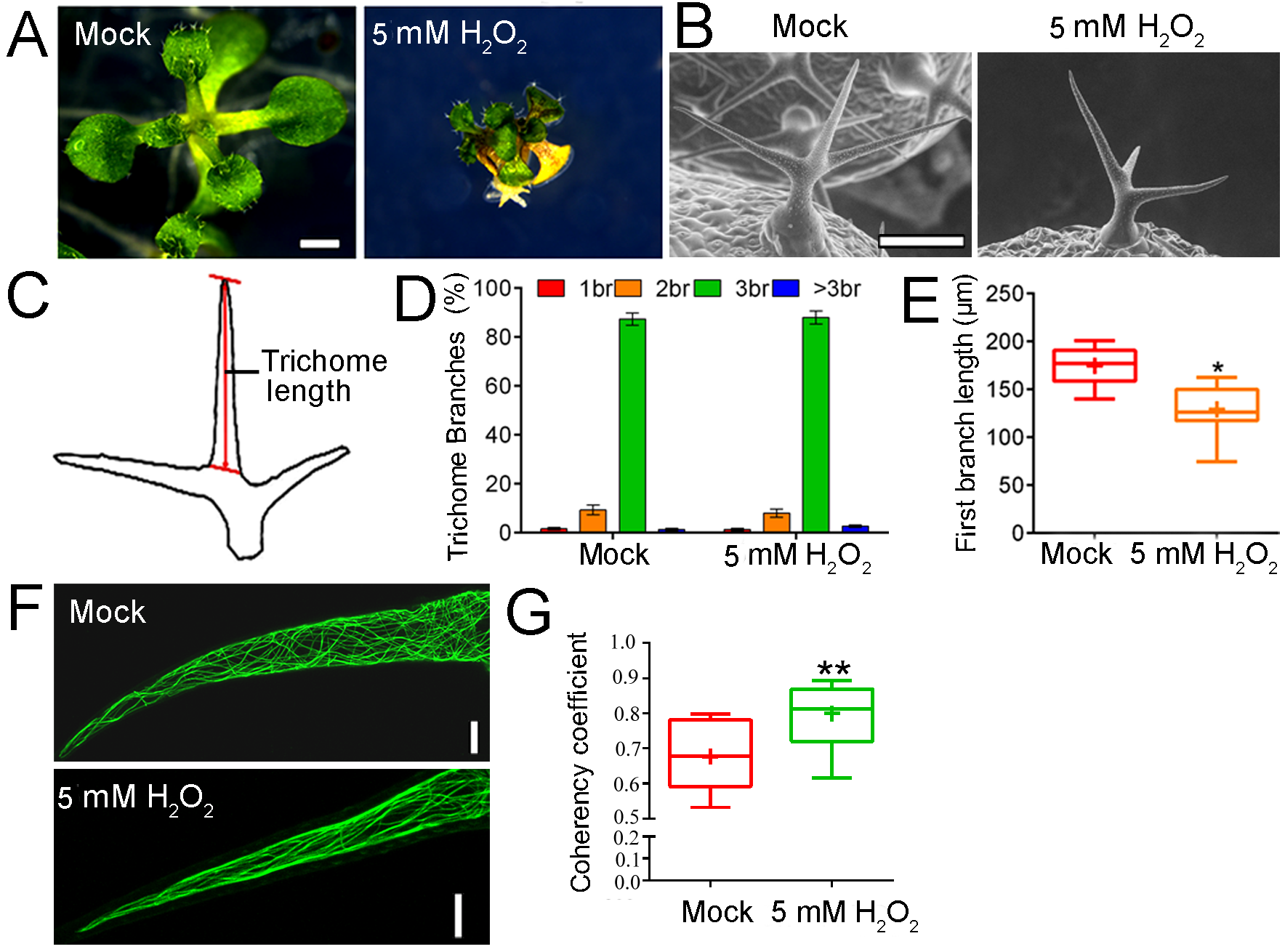

Supplement: S17 Fig — (A) Representative images of 15-day-old seedlings with or without H2O2 treatments. WT seeds were sterilized and then grew on Murashige and Skoog medium agar plates supplemented with 0 and 5 mM H2O2, respectively. 15-day-old seedlings were used for phenotype analyses. Scale bar = 1 mm. (B) Representative images of trichomes from 15-day-old seedlings with or without H2O2 treatments. WT seeds were sterilized and then grew on Murashige and Skoog medium agar plates supplemented with 0 and 5 mM H2O2, respectively. 15-day-old seedlings were used for analysis of trichome phenotype. Scale bar = 100 μm. (C) A cartoon depicting how the trichome length was measured. (D) Trichome branch (br) distribution of mock and H2O2 treatment. Values are given as the mean ± SD. (E) Quantification of the trichome length. Mann–Whitney U test, *P < 0.05 (P = 0.04255). Values are given from more than 30 trichomes. (F) Visualization of cortical microtubules in trichomes from 15-day-old seedlings with or without H2O2 treatments. Seeds of a transgenic line stably expressing GFP-MBD were sterilized and then grew on Murashige and Skoog medium agar plates supplemented with 0 and 5 mM H2O2, respectively. Trichomes of 15-day-old seedlings were used for imaging analyses. Scale bar = 10 μm. (G) Quantification of microtubule alignment. The microtubule alignment measurement was carried out with "OrientationJ", a ImageJ plug-in, to calculate the directional coherency coefficient of the fibers. A coherency coefficient close to 1 represents a strongly coherent orientation of the microtubules. For the boxplots, the box extending from the lower to upper quartile values of the data, with a line representing the data medians. The whiskers extending past 1.5 of the interquartile range. Mann–Whitney U test, **P < 0.01 (P = 0.00393). Values are given from more than 20 trichomes. (TIF) [file pgen.1007705.s017.tif]

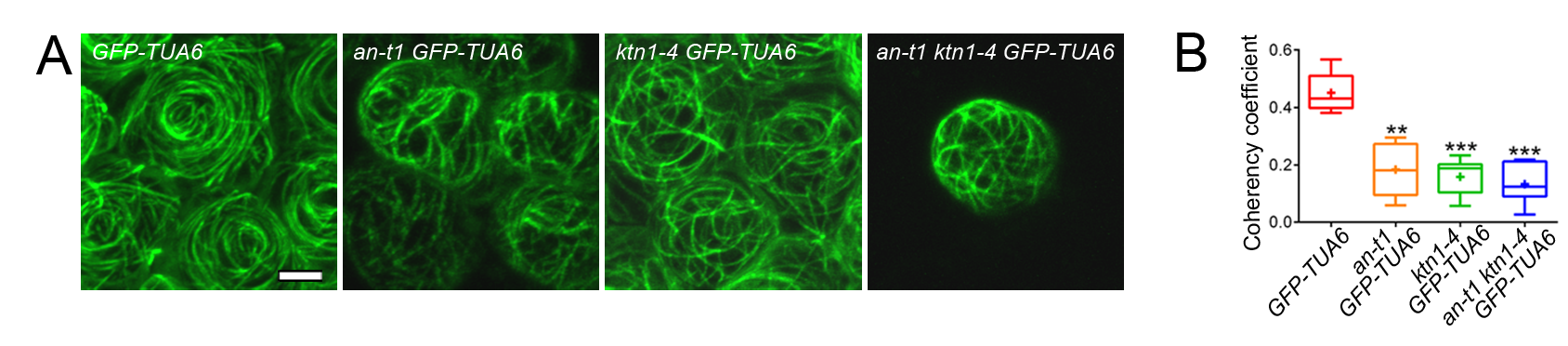

Supplement: S18 Fig — (A) Visualization of microtubules in conical cells from mature petals. Representative confocal images were generated via surface projections of image stacks at 0.5-μm intervals from the top view of adaxial epidermis of non-folded petals. The microtubule reporter line stably expressing GFP-TUA6 was used for the observation of microtubule arrays. Scale bar = 5 μm. (B) Quantification of microtubule alignment. The microtubule alignment measurement was carried out with "OrientationJ", a ImageJ plug-in, to calculate the directional coherency coefficient of the fibers. A coherency coefficient close to 1 represents a strongly coherent orientation of the microtubules. Mann–Whitney U test, **P < 0.01, ***P < 0.001 (from left to right, P = 0.00759, P = 0.0002, P = 0.00088). Values are given from 40 cells of 10 petals. (TIF) [file pgen.1007705.s018.tif]

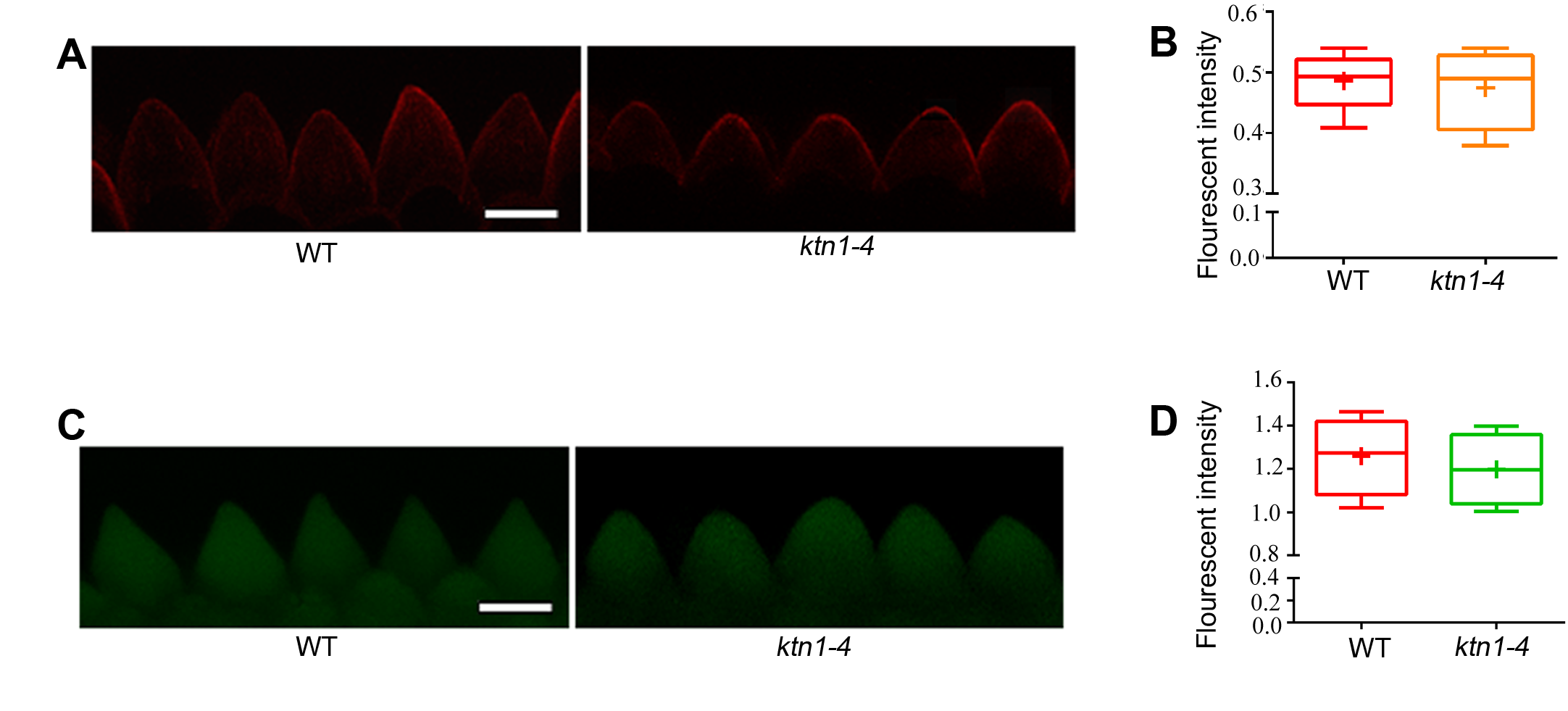

Supplement: S19 Fig — (A) DHE-stained conical cells for O2• – analysis of WT and ktn1-4 from stage 14 flowers. Scale bars = 10 μm. (B) Quantification of DHE-detected O2• – in conical cells. For comparative O2• – analysis, a region of interest (ROI) at the conical cells was quantified by ImageJ. For the boxplots, the box extending from the lower to upper quartile values of the data, with a line representing the data medians. The whiskers extending past 1.5 of the interquartile range. ns indicating no significant difference from WT (Mann–Whitney U test, P > 0.05). Values are given from 140 cells of 20 petals. (C) CM-H2DCFDA stained conical cells for H2O2 analysis of WT and ktn1-4 from stage 14 flowers. Scale bars = 10 μm. (D) Quantification of CM-H2DCFDA-detected H2O2 in conical cells of WT and ktn1-4. For comparative O2• – analysis, a region of interest (ROI) at the conical cells was quantified by ImageJ. ns indicating no significant difference from WT (Mann–Whitney U test, P > 0.05). Values are given from 140 cells of 20 petals. (TIF) [file pgen.1007705.s019.tif]
