# Supplementary material for: Reactive oxygen species mediate conical cell shaping in Arabidopsis thaliana petals
Source: PLoS Genet. 2018 Oct 8;14(10):e1007705. doi: 10.1371/journal.pgen.1007705 (PMC6203401; doi:10.1371/journal.pgen.1007705)
Supplement: S2 Table — (DOCX) [file pgen.1007705.s021.docx]

**S2 Table. The primary primers used in this work**

| Primer name | Sequence |  |
| --- | --- | --- |
| KTN1-QRT-F | CTTCTTCAATGTATCTTCTGCTA |  |
| KTN1-QRT-R | AGTTCCGATTTCACCCTT |  |
| KTN1-RT-F | CCGTTGGGATGATGTTGCAGGTT |  |
| KTN1-RT-R | TGTCGCTGTATTGCTCACTCCATC |  |
| pKTN1-U | GGATCCTTCTGTTTTCTTCTACTCTC |  |
| pKTN1-L | ACATGTTTCCTCTTTTACTAAAAAAATAG |  |
| KTN1-pGWB504-U | GGGGACAAGTTTGTACAAAAAAGCAGGCTCCTTCTGTTTTCTTCTACTCTC |  |
| KTN1-pGWB504-L | GGGGACCACTTTGTACAAGAAAGCTGGGTTAGCAGATCCAAACTCAGAG |  |
| KTN1-CDS-F | GGGGACAAGTTTGTACAAAAAAGCAGGCTTG ATGGTGGGAAGTAGTAATTCG |  |
| KTN1-CDS-R | GGGGACCACTTTGTACAAGAAAGCTGGGTGAGCAGATCCAAACTCAGAG |  |
| AN-RT-F | GCTGTTGGATGATTGTGCTGTGA |  |
| AN-RT-R | CCTGTTGCCTACTGGTGGATTC |  |
| pAN-U | AAGCTTTTCTACGTCCGTATCGCTC |  |
| pAN-L | TCTAGACTCCTCCTTCTTCTCGCTACTC |  |
| AN-CDS-U | ATGAGCAAGATCCGTTCGTC |  |
| AN-CDS-L | TTAATCGATCCAACGTGTGATAC |  |
| CAT3-CDS-F | GGGGACAAGTTTGTACAAAAAAGCAGGCTTGATGGATCCTTACAAGTATCG |  |
| CAT3-CDS-R | GGGGACCACTTTGTACAAGAAAGCTGGGTGGATGCTTGGCCTCACGTTC |  |
| CAT2-CDS-F | GGGGACAAGTTTGTACAAAAAAGCAGGCTTGATGGATCCTTACAAGTATCG |  |
| CAT2-CDS-R | GGGGACCACTTTGTACAAGAAAGCTGGGTGGATGCTTGGTCTCACGTTCA |  |
